# BMP Signaling Mediates Effects of Exercise on Hippocampal Neurogenesis and Cognition in Mice

**DOI:** 10.1371/journal.pone.0007506

**Published:** 2009-10-20

**Authors:** Kevin T. Gobeske, Sunit Das, Michael A. Bonaguidi, Craig Weiss, Jelena Radulovic, John F. Disterhoft, John A. Kessler

**Affiliations:** 1 Department of Neurology, Northwestern University's Feinberg School of Medicine, Chicago, Illinois, United States of America; 2 Department of Neurosurgery, Northwestern University's Feinberg School of Medicine, Chicago, Illinois, United States of America; 3 Department of Physiology, Northwestern University's Feinberg School of Medicine, Chicago, Illinois, United States of America; 4 Department of Psychiatry, Northwestern University's Feinberg School of Medicine, Chicago, Illinois, United States of America; Peking University, China

## Abstract

Exposure to exercise or to environmental enrichment increases the generation of new neurons in the adult hippocampus and promotes certain kinds of learning and memory. While the precise role of neurogenesis in cognition has been debated intensely, comparatively few studies have addressed the mechanisms linking environmental exposures to cellular and behavioral outcomes. Here we show that bone morphogenetic protein (BMP) signaling mediates the effects of exercise on neurogenesis and cognition in the adult hippocampus. Elective exercise reduces levels of hippocampal BMP signaling before and during its promotion of neurogenesis and learning. Transgenic mice with decreased BMP signaling or wild type mice infused with a BMP inhibitor both exhibit remarkable gains in hippocampal cognitive performance and neurogenesis, mirroring the effects of exercise. Conversely, transgenic mice with increased BMP signaling have diminished hippocampal neurogenesis and impaired cognition. Exercise exposure does not rescue these deficits, suggesting that reduced BMP signaling is required for environmental effects on neurogenesis and learning. Together, these observations show that BMP signaling is a fundamental mechanism linking environmental exposure with changes in cognitive function and cellular properties in the hippocampus.

## Introduction

Neural stem cells and their progenitor cell progeny persist throughout life in two specialized niches of the adult brain: the subgranular zone (SGZ) of the hippocampal dentate gyrus and the subventricular zone (SVZ) of the lateral ventricles. The unique properties of these cells and the niche substrate combine to enable the localized propagation and maturation of neural progenitor lineage species [1]–[3]. Adult neurogenesis is a multi-step process involving cell proliferation, differentiation, survival and integration into functional circuits [4]–[6], and it can be regulated at many stages. Exposure to exercise or environmental enrichment each affects multiple aspects of cell progression in the SGZ/SVZ niches, leading to enhanced neurogenesis and cognition associated with these areas [7]–[11]. Activity-dependent regulation of neurogenic niche properties may even be essential for normal behavioral adaptation to a changing environment [12]–[15]. In particular, the integration and turnover of new cells within established networks may contribute to the coding of cortical input into the hippocampal tri-synaptic circuit [16]–[21].

In addition to being a site for ongoing neurogenesis, the dentate gyrus (DG) has many unique characteristics within the hippocampus. The DG is comprised of tightly packed, radially organized granule cells, similar to the layers of the cerebellum and neocortex also receiving ascending input from other brain regions. Dentate granule cells receive cholinergic transverse septal fibers from the basal forebrain, glutamatergic medial/lateral perforant path projections from the entorhinal cortex, and GABAergic input from medial septum and local interneurons [22]. DG granule cells integrate these signals, and send their own mossy fiber axons to the CA3 region of the hippocampus as part of the classical DG-CA3-CA1 tri-synaptic pathway. Granule cells vastly outnumber the fibers they receive, creating very sparse firing patterns feeding into the rest of the hippocampal circuit. This increases the discriminative capacity of the network, since minor differences amongst similar patterns become separated or “orthogonalized” in their coding [17]–[21]. The cycling of young cells into the circuit also adds a temporal dimension to the coding substrate, which may be essential for the functions of coincidence detection and sequence separation performed by the DG [17]–[19]. Young cells have lower excitation thresholds and higher amplitude LTP than their mature neighbors. This effectively subdivides the set of cells receiving and processing input at this step in the circuit, adding another layer in its capacity to discriminate novel information [23]–[26]. While young cells can receive tonic and synaptic input from multiple transmitters, and can project similar outputs even before their terminal division, with time their electrophysiological properties come to match those of mature ones [3], [6], [27], [28]. However, their continued replenishment maintains a ratio between the two cell populations that is itself an integral property of the coding substrate [17]–[21], [25], [26]. It is not clear whether the functional regulation of the network occurs at the level of changing ratios of excitable populations, of individual cells representing new memories, or a combination of the two.

The precise role of neurogenesis in hippocampal cognition has also been difficult to determine. The diversity of methods used to ablate proliferation [12]–[15], [29]–[32] in varied strains of mice [32]–[35] and to assess cognitive behavior on multiple “standard” testing instruments [36] has led to mixed results and interpretations. Yet such differences may be clarified by recent studies illuminating the functions of distinct hippocampal subfields [37]–[39], the lineage-wise progression of different transmitter types and actions in new cells [40]–[43] and the application of specific behavioral tests [20], [38], [39], [44]–[48]. Cognitive tasks which involve multiple subfields or emphasize processing outside the DG may not optimally reflect the functional role of SGZ niche properties [37]–[48]. Traditional versions of the Morris water maze may employ spatial coding functions of the CA3 and CA1 subfields more than the DG [38], [39], [49]. Contextual fear conditioning incorporates input from the lateral amygdala into CA1 [50], as well as from the entorhinal cortex into the DG [34], [51]. Paradigms which reduce the proportion of non-DG coding often show a stronger correlation between SGZ niche properties and learning, and will be the foundation for new tests to clarify such mechanisms. This correlation is also influenced by the method to reduce levels of neurogenesis. Commonly, external factors such as irradiation [13], [14], [31], [33], [34], [51]–[55], anti-mitotic drugs [12], [29], [30], [56], or induction of toxin sensitivity [34], [51], [57]–[59] are used to kill dividing cell populations. Fewer studies have targeted pathways with proposed roles in the physiological regulation of the neurogenic niche, aiming to reduce neurogenesis as a process rather than an outcome [60]–[64]. Growth factors such as brain-derived neurotrophic factor (BDNF), vascular endothelial growth factor (VEGF) and insulin-like growth factor 1 (IGF-1) are activity-dependent modulators of niche properties and are important potential tools in the study of functional regulation. Transgenic knockdown of these factors [65]–[67] or their receptors [68]–[70], or treatment with blocking antibodies [71]–[73], can reduce baseline or exposure-mediated levels of neurogenesis and cognitive performance. Yet, few interventions describe a mechanism capable of bi-directionally blocking or reproducing the effects of exercise on neurogenesis and cognition, as might be predicted for the physiological processes at hand.

Bone morphogenetic protein (BMP) signaling is an important regulator of cell proliferation and fate commitment throughout development and within the adult SVZ and SGZ neurogenic niches [74]–[79]. Binding of BMPs to their receptors initiates phosphorylation and nuclear translocation of SMAD1/5/8, prompting transcription of target genes for cell cycle exit and astrocytic fate commitment [80]–[83]. BMP actions are regulated *in vivo* by a family of cysteine knot proteins including noggin, chordin and follistatin that competitively bind BMPs in the extracellular space, preventing receptor activation and all downstream signaling activity [84], [85]. BMP4 is one of the primary BMP family members in the adult neurogenic niche and noggin is a main BMP inhibitor [86], [87]. Inhibition of BMP signaling by noggin maintains the stem cell pool and the numbers of neural progenitor cells (NPCs) and oligodendrocyte progenitor cells in the SVZ [76], [88]. Noggin also promotes the proliferation and differentiation of adult hippocampal NPCs both *in vivo* and *in vitro* [78], [79]. However the regulation of BMP signaling by activity has not been determined.

Since ongoing BMP signaling appears to be important for the regulation of the hippocampal neurogenic niche, we hypothesized that it might also mediate the effects of exercise exposure on hippocampal neurogenesis and cognitive functioning. We found that exercise reduced hippocampal BMP signaling levels in a coordinated manner before the onset of cognitive improvements. Blocking BMP signaling by either transgenic or pharmacological methods reproduced the effects of exercise on learning and neurogenesis. Transgenic overexpression of BMP4 prevented the actions of exercise on both cognition and lineage regulation of the SGZ niche. Together these observations suggest that BMP signaling is a fundamental mechanism linking experience, behavior and cellular lineage properties in the hippocampus.

## Results

### Elective exercise decreases BMP signaling in the hippocampus prior to enhancement of hippocampus-dependent cognitive functioning

The effects of exercise on hippocampus-dependent learning and hippocampal neurogenesis are not immediate, but require a sufficient duration of exposure. Such a lag period suggests a mechanism in which regulation of a signaling pathway initiates cellular changes that may then support behavioral adaptations. To establish whether BMP signaling is regulated by exercise, and could be an activity-dependent determinant of learning and neurogenesis, we compared the time course for changes in the levels of hippocampal BMP signaling molecules, cellular proliferation and cognitive performance. We exposed separate groups of 2 month old C57Bl/6 mice to running wheels for 0,2,4,7 or 10 days. For each exposure group, we used qRT-PCR to measure levels of mRNA for BMP4 and noggin, the primary BMP effectors in the hippocampus. Since changes in mRNA levels do not directly indicate changes in functional signaling proteins, we performed Western blot analysis of BMP4 and noggin proteins in separate groups of mice exposed to running for 0,4,7,10 or 14 days. We also measured levels of proliferating cell nuclear antigen (PCNA) as a marker for changes in rates of cell division. To determine the temporal association between changes in BMP signaling and cognitive performance, we evaluated Y-maze scores for spontaneous alternation behavior in each group of mice.

**Figure 1:**
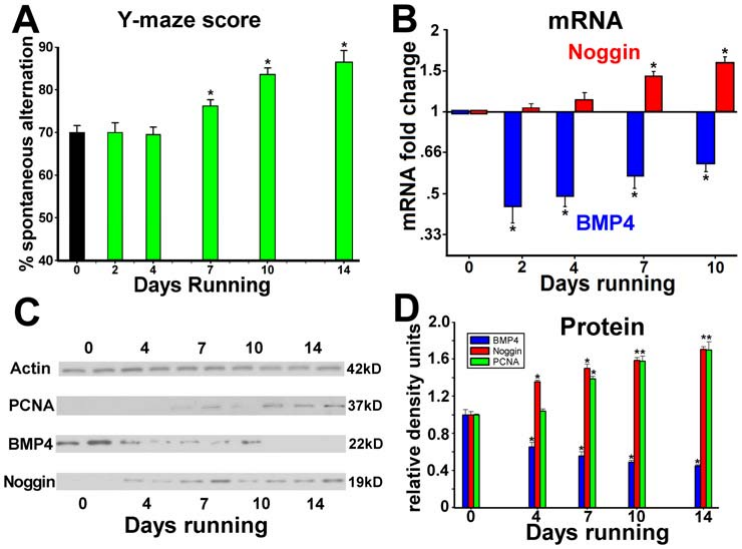
Elective exercise decreases BMP4 and increases noggin expression in the hippocampus. Separate groups of C57Bl/6 mice were exposed to running wheels for 0, 2, 4, 7, 10 or 14 days (n = 8 per exposure group). (A) Performance on a Y-maze task of hippocampus-dependent cognition shows a duration-response curve with exercise-induced improvements beginning after 7 days of running exposure. (B) qRT-PCR analyses show decreases in levels of BMP4 mRNA and increases in noggin mRNA in the hippocampus preceding the onset of improvements in Y-maze performance. (C) Examples of Western blots for noggin, BMP4 and PCNA protein in the hippocampus after increasing durations of exposure to elective exercise. (D) Quantification of the Western analyses of hippocampal BMP4, noggin and PCNA shows a duration-dependent relationship, with the onset of changes in BMP4 and noggin levels preceding those for the cell proliferative marker, PCNA. * Differs from control (day 0) group at p<0.01

Exercise improved Y-maze performance in most mice by the 7^th^ day of exposure, with further increases after 10 days for all mice tested (Fig. 1A) (p<.01). After only 2 days of running, BMP4 mRNA was reduced to less than half of naïve levels and it remained decreased for all subsequent time-points (p<.01). Levels of noggin mRNA did not change until 4 days, but had increased 1.5-fold by 7–10 days of exercise (Fig. 1B) (p<.001). Thus mRNA for BMP4 was decreased and mRNA for noggin was increased before and during changes in Y-maze performance. After normalization to actin loading controls, densitometric levels of BMP4 protein were reduced by 35% after 4 days, and by nearly 50% after 7 days exposure, where they remained thereafter. Relative to naïve levels, hippocampal noggin protein significantly increased by 36% after 4 days and remained elevated by 50% after 7 days. Hippocampal levels of PCNA were increased above baseline by 39% by 7 days, and remained elevated for longer running durations (Fig. 1C,D). This increase in PCNA occurred after changes in levels of noggin and BMP4, but before gains in Y-maze performance. Running also reduced hippocampal levels of the marker of activated BMP signal transduction, pSMAD1/5/8, and particularly decreased the number of cells with strong nuclear (npSMAD1/5/8) staining in the SGZ, further supporting the spatial concurrence of these findings (Fig. S1). Thus, exercise decreased production of BMP4 and increased levels of the BMP inhibitor, noggin, in advance of gains in cell proliferation and Y-maze performance. The complementary changes in levels of BMP4 and noggin suggest a conserved physiological process by which down-regulation of BMP signaling precedes the effects of environmental exposure on the cellular properties of the neurogenic niche.

### Transgenic manipulation of BMP signaling in NSE-BMP4 and NSE-noggin mice models the regulation of the neurogenic niche by physical activity

The observation that exercise exposure leads to reductions in hippocampal BMP signaling before and during improvements in cognition suggests that down-regulation of BMP signaling might mediate the effects of the environment on hippocampal niche properties and behavior. To determine the role of BMP activity in hippocampal cognition, it was necessary to manipulate BMP signaling separately from its regulation by environmental stimuli. Since constitutive knockout or overexpression of BMP4 has major developmental consequences, we placed expression of BMP4 or its inhibitor, noggin, under temporal and tissue-specific control of the promoter for neuron specific enolase (NSE) as described previously [78], [89], [90]. NSE production first begins at low levels antenatally in forebrain neurons, but is not expressed during the period of developmental neurogenesis and establishment of cortical and hippocampal structures. During young adulthood, NSE production increases to reach high levels in the forebrain and hippocampus, with expression primarily maintained in the hippocampus thereafter [89], [91]. Founder NSE- transgenic mice were backcrossed 5–7 generations onto the C57Bl/6 background strain, which have appropriately high baseline levels of neurogenesis and reliable cognitive performance [7], [32], [92]. Thus, any changes in the cellular or cognitive phenotypes of the NSE-noggin and NSE-BMP4 mice can be compared against wild type animals exhibiting functionally salient measures of behavior, cell signaling and histology.

**Figure 2:**
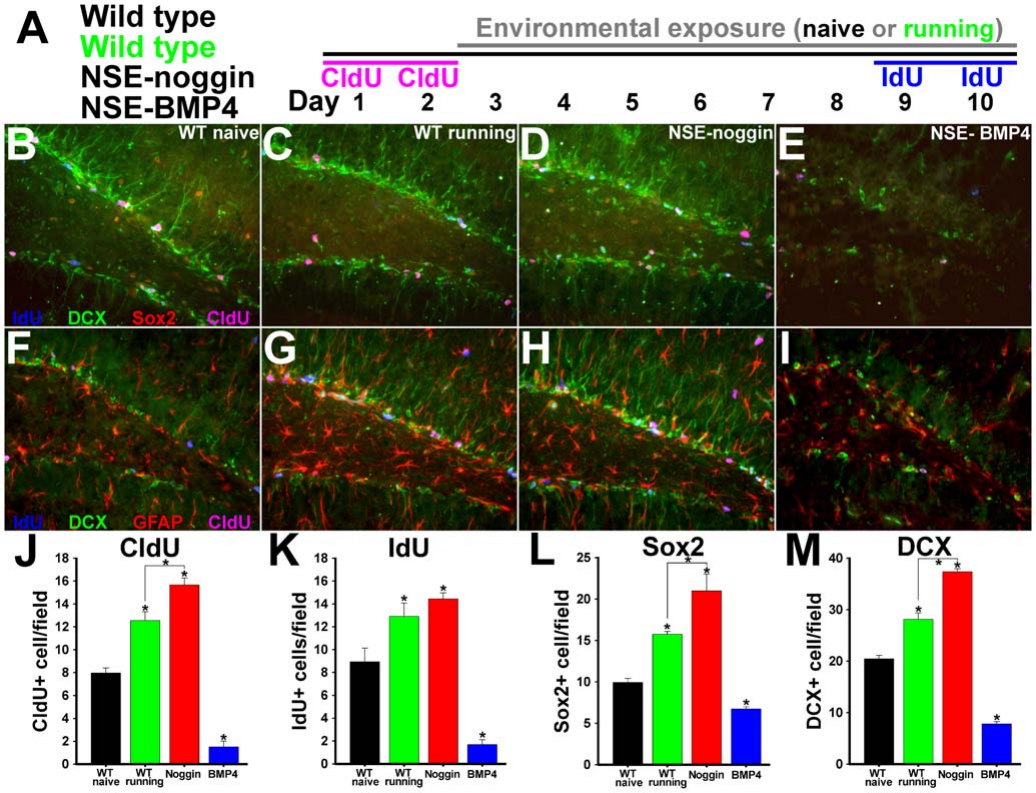
Transgenic regulation of BMP signaling mirrors the effects of exercise on SGZ niche cell lineage properties. (A) Schematic of the experimental design. All 4 groups of animals (N = 8 per group) received CldU for 2 days. One group of wild type animals was exposed to elective exercise for 8 days while the other groups were maintained in standard housing. On the last 2 days all 4 groups of animals received IdU. Coronal sections through the dentate gyrus were then immunostained for DCX (green), CldU (pink), IdU (blue), and Sox2 (red: panels B–E) or GFAP (red: panels F–I). Panels B–E and F–I show representative merged images for all sets of four labels. Individual channels comprising merged images are shown in Figure S2. (B,F) Wild type, standard housing; (C,G) Wild type, running; (D,H) NSE-noggin. (E,I) NSE-BMP4. (J–M) Unbiased stereological sampling and quantification per 40X field of cells labeled for (J) CldU; (K) (IdU); (L) Sox2; (M) DCX. Analyses of double labeling are shown in Figure 3. Analysis of coronal sections labeled for nestin is presented in Figure S3. * Differs from wild type naïve group at p<0.001 or from other groups as indicated at p<0.01 by Bonferroni/Dunn pair-wise comparison.

Mice overexpressing BMP4 were previously shown to have decreased cell proliferation and stem/progenitor cell numbers in the dentate gyrus, whereas noggin transgenic mice have increased numbers of neural progenitor cells relative to wild type animals [78], [79]. However NSE- transgenic mice show no differences in the overall structure of the hippocampus or the thickness of the DG granule cell layer (GCL) relative to WT controls [79], [89]. To determine the effects of BMP signaling on the SGZ niche, we developed a technique to examine pairs of markers for cell division and progenitor identity, with respect to progression through time and the SGZ lineage, in NSE-noggin, WT running, WT naïve and NSE-BMP4 mice. The thymidine analogs, chloro-deoxyuridine (CldU) or iodo-deoxyuridine (IdU), were administered at separate times to label cell division before or after exposure to exercise or standard conditions (outlined in Figure 2A). CldU and IdU are incorporated during DNA synthesis and are labeled by specific antibodies equivalently to BrdU. SGZ cell lineage identity was also labeled with Sox2 or GFAP to mark neural stem cells (NSCs), nestin to mark early progenitors, doublecortin (Dcx) to mark neuroblasts and young neurons, or NeuN to mark mature neurons. All slides were labeled using paired combinations of markers for earlier/later lineage identity and for CldU/IdU birth-dating. This demonstrates both the stage within the stem cell lineage and the time since cell division for numerous cells within the same section (Fig. 2B–I). Each slide is thus a comprehensive snapshot of niche progression and regulation during exercise or BMP manipulation. Individual channels comprising merged images in Figure 2 are shown in Figure S2.

**Figure 3:**
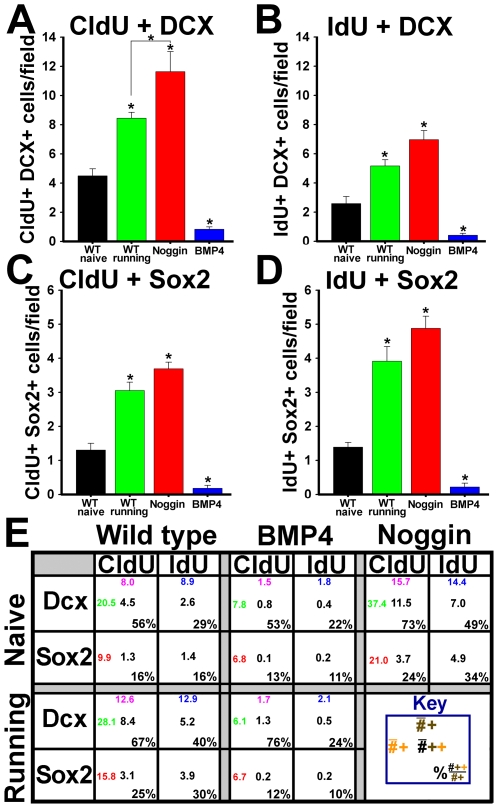
Exercise and inhibition of BMP signaling promote proliferation of multiple SGZ progenitor subtypes. (A–D) Unbiased stereological sampling of coronal sections through the hippocampus and quantification of cells co-labeled with (A) CldU and Dcx, (B) IdU and Dcx, (C) CldU and Sox2, and (D) IdU and Sox2. (E) Table detailing the early/late progenitor identity of cells dividing at different points in time for different genotypes and exposure to running. The number in pink on the top is the number of CldU labeled cells per field, the blue number on the top is the number of IdU labeled cells, the number in red on the left is the number of Sox2 labeled cells, and the number in green on the left is the number of DCX labeled cells. Black numbers indicate cells marked for division which are also labeled as a given progenitor subtype. Percentages indicate the fraction of dividing cells within a given lineage species for each group of mice. * Differs from wild type naive group at p<0.01. The marked reductions in the number of proliferative Sox2-labeled and Dcx-labeled cells in the NSE-BMP4 mice indicates that BMP signaling acts on both early and later populations of precursors. The increase in both CldU and IdU labeling of the Dcx labeled cells in the NSE-noggin and WT running mice suggests that inhibition of BMP signaling increases proliferation of later precursors as well as earlier ones.

The number of cells labeled with Dcx, Sox2, CldU or IdU were all significantly increased in running-exposed WT mice and NSE-noggin mice, whereas numbers were markedly depleted in NSE-BMP4 animals (Fig. 2J–M). Analysis of cells co-labeled with IdU or CldU and markers of progenitor subtype was used to determine the influences of exercise and BMP signaling on the point of cell cycle reentry along the neurogenic lineage (Fig. 3A–D). Such patterns were nearly identical for NSE-noggin and running WT mice relative to WT naïve controls, suggesting a similar mechanism of niche regulation. The number of cells positive for both CldU + Dcx, or CldU + Sox2, increased 1.9-fold or 2.3-fold in running WT mice relative to naïve controls (p<.01). Such counts were 2.6-fold or 2.8-fold higher in NSE-noggin mice compared with naïve WT animals (Fig. 3A,B,E) (p<.01). Conversely, these cell populations in NSE-BMP4 mice were reduced by 81% or 86%, respectively. Numbers of cells positive for both IdU + Dcx, or IdU + Sox2, increased 2-fold or 2.8-fold in running WT mice (p<.01), and were 2.7-fold or 3.5-fold higher in NSE-noggin mice relative to controls (Fig. 3C–E) (p<.01). Both populations of cells in NSE-BMP4 mice were reduced by 84%. Interestingly, noggin expression and exercise exposure increased cell cycle entry of both early and later NPCs, suggesting a progressive system of functional niche regulation. The table provided in Figure 3E describes these counts and the proportions of CldU+ or IdU+ cells expressing given lineage markers in detail.

Within the SGZ stem cell lineage, glial fibrilary acidic protein (GFAP) and Sox2 mark similar populations of relatively quiescent NSCs [4]–[6], [93]. As with counts for Sox2 above, numbers of GFAP+ cells in the SGZ were increased in WT running and NSE-noggin mice, whereas those for NSE-BMP4 mice were reduced relative to controls (Fig. 2F–I). Further, the morphology of such cells was notably regulated by exercise and BMP signaling, with bipolar, radial cell types abounding in NSE-noggin and WT running mice. Stellate, multi-polar GFAP+ cells prevailed in NSE-BMP4 mice, consistent with increased pro-astrocytic fate commitment as in previous reports [78], [79]. Populations of cells expressing nestin, which marks stem-like and early progenitor cells prior to the onset of pro-neuronal identity and Dcx expression [4]–[6], were regulated similarly to levels of GFAP and Sox2 (Fig. S3). Numbers of nestin+ cells were increased by 42% and 89% in WT running and NSE-noggin mice (p<.01), whereas nestin+ cells were decreased by 71% in NSE-BMP4 mice relative to WT controls (p<.01). Compared with the effects on SGZ progenitors, running and BMP signaling did not alter mature populations labeled with NeuN. Consistent with the lineage progression of IdU+Dcx+ cohorts above, exercise and noggin increased the subset of NeuN+ cells that had divided as late NPCs 10 days before staining (CldU+) (Fig. S3B–G). Yet noggin did not increase the percentage of CldU+ cells that had progressed toward a mature neuronal identity. This suggests that reduced BMP signaling mirrors the effects of exercise on mitotic cell populations, but other activity-dependent mechanisms may affect neuronal survival and integration (Fig. S3C,H). Thus, running and inhibition of BMP signaling both promote cell cycle reentry at multiple points along the neural lineage, leading to increased neurogenesis.

### Transgenic changes in BMP signaling reproduce the effects of physical activity on learning and memory

Since transgenic decrease in BMP signaling mimicked the effects of exercise on hippocampal niche properties (Fig. 2), we hypothesized it might also mirror the effects of exercise on hippocampal cognition. We therefore examined multiple aspects of hippocampal cognition in the NSE-noggin and NSE-BMP4 mice relative to WT controls using an array of tests of different length and complexity. Tests covered 4 hippocampal learning modalities, including spontaneous alternation, recognition and discrimination, associative memory and spatial learning. Tests were further designed to preclude confounding by stress or motor behavior, and to highlight the role of the dentate gyrus, where transgenic changes in BMP signaling are the greatest [79]. Numerous control tests for hippocampus-independent functioning were also performed. Running-induced gains have been confirmed in WT mice for all tests described [7], [8], [54], [63], [67], [71]–[73], [94]–[97].

#### Spontaneously Alternating Exploration

**Figure 4:**
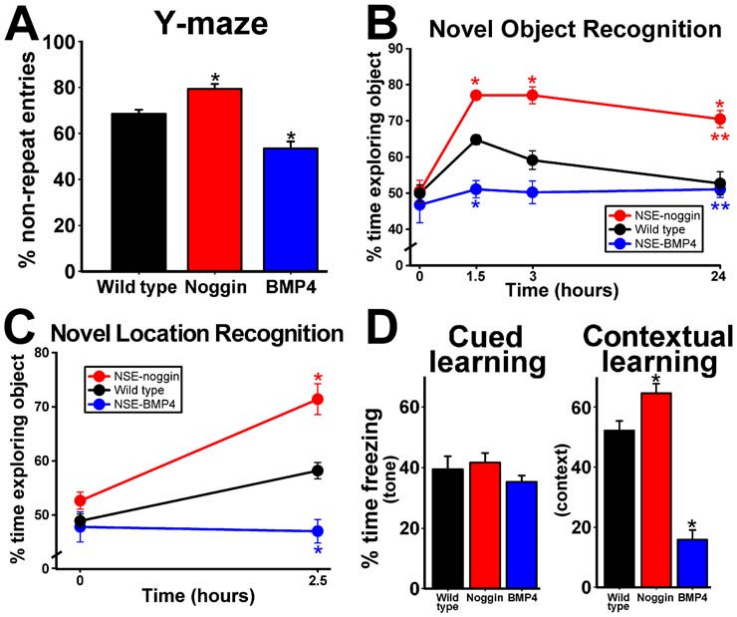
BMP signaling regulates cognitive performance on hippocampus-dependent behavioral and memory tasks. Wild type, NSE-noggin, and NSE-BMP4 mice (n = 10 per group per experiment) were tested on a series of learning and memory tasks to measure baseline levels of hippocampal cognitive performance, processing and recall. (A) Performance on a Y-maze test measuring spontaneous alternation as a hippocampus-dependent exploration strategy shows significant gains in NSE-noggin mice and significant deficits in NSE-BMP4 mice relative to wild type controls. (B) NSE-noggin mice have significantly improved object memory on the novel object recognition test after multiple delay periods, and (C) significantly better spatial memory on the novel location recognition test relative to wild type animals. NSE-BMP4 mice are impaired on all tests, never significantly exceeding chance levels of performance. (D) Fear conditioning tests for hippocampus-independent cued memory and hippocampus-dependent contextual memory show that the cognitive phenotypes and the effects of BMP signaling are specific to the hippocampus. All mice perform equally on the cued memory test, while the percentage of time freezing on the contextual memory test is increased for NSE-noggin mice and decreased for NSE-BMP4 mice relative to wild type controls. * Differs from wild type group at p<0.01 by Bonferroni/Dunn pair-wise comparison. **Significant main effect by multiple ANOVA for group x delay comparisons with all groups differing from each other at p<0.05.

To measure the effect of transgenic manipulation of BMP signaling on hippocampal cognitive performance, NSE-noggin, wild type and NSE-BMP4 mice were probed on a single-trial, three-armed, Y-maze test of hippocampus-dependent processing (Fig. 4A) [98]. Efficient exploration of this novel environment requires the fewest entries into previously visited arms. Thus, any triad of sequential arm-entries should have no repeats, referred to as “spontaneous alternation.” During a 5-minute free exploration period, NSE-noggin mice had significantly fewer reentries into arms visited immediately prior to the occupied arm, indicating improved exploration and hippocampal processing (Fig. 4A). Mice overexpressing BMP4 were significantly impaired relative to wild type controls, exploring the maze in an essentially random fashion.

#### Feature Recognition and Discrimination

We next tested for differential abilities in hippocampus-dependent recall using the Novel Object Recognition (NOR) and Novel Location Recognition (NLR) tests. For the NOR and NLR tests, mice were extensively habituated to an open field for two days prior to testing. During the first “exposure” trial of the test, mice were placed back into the familiar field, but which now contained two identical objects in bilateral mirror-image orientation. Mice investigated the testing arena until they amassed 30 seconds of object-exploration time. After a given delay period (1.5, 3 or 24 hours for different groups), one of the items was exchanged for a novel object (NOR) or was placed in a novel location within the field (NLR), and the mice were reintroduced into the arena for the “recall” trial. Mice have a natural predilection for novelty, so the fraction of cumulative object-exploration time spent at the new object or location indicates the degree of memory for the familiar stimulus. Since this test requires contextual and feature discrimination relative to spatial orientation and time, it is strongly hippocampus-dependent, with emphasis on the DG [46]–[48]. NSE-noggin mice had significantly better scores for both object and location memory (Fig. 4B,C) compared with controls. NSE-BMP4 mice performed at levels equivalent to random chance. Remarkably, separate groups of NSE-noggin mice showed strong discrimination of the novel object even after a delay of 3 or 24 hours. Memory in wild type mice showed declines after a 3 hour wait, and had reached chance levels by 24 hours.

#### Associative Learning

To test the hippocampal specificity of such cognitive findings more directly, we performed a fear conditioning paradigm for cued and contextual recall of an aversive stimulus. Measures for general activity during exposure to the testing chamber, and for startle responses upon presentation of the tone (conditioned stimulus, CS) and the shock (unconditioned stimulus, US) were equivalent for all mice (Fig. S4A,B). Mice also showed similar patterns of habituation to the CS when no longer paired with the US during the course of the recall trial (Fig. S4C). However, on the hippocampus-dependent contextual conditioning task, NSE-noggin mice showed significantly more freezing behavior upon re-introduction to the exposure chamber 24 hours after the context-US pairing (Fig. 4D). NSE-BMP4 mice were significantly impaired on this measure of associative learning. All mice froze equally upon re-exposure to the cued tone stimulus. This suggests all mice possessed similar hippocampus-independent learning abilities, and that BMP signaling modulates hippocampal cognition without altering global or extra-hippocampal function. Notably, our conditioning paradigm used only a single, mild CS-US pairing, so as not to saturate the role of amygdala-dependent functions, and to increase the discrimination of hippocampus-dependent vs. –independent aspects of the task.

#### Spatial Reference Learning and Adaptation

Although the Morris water maze remains the standard test of murine spatiotemporal learning, recent studies have questioned its confounding by stress, relevance to DG processing, and its ethological validity [36], [99]–[101]. A new paradigm moving the platform every day was designed to mitigate some of these concerns (see Methods). By this strategy, mice must integrate novel episodic information within the overarching context of spatial reference learning. This schema emphasizes the role of the dentate gyrus in pattern separation, relative to the role of whole circuit in spatial-relational information processing [44]–[49], [102]. Since stress may affect adaptive learning more than incremental reference learning, comparisons between these measures clarify affective behavioral influences. Maze training occurred over 5 days, with 4 testing trials per day. Each day the platform was moved to a new target location within the pool, and mice were given a 15-second “primer” exposure to the new location two hours before the first daily trial. This period between the primer and the start of trial-by-trial training thus requires the rapid adaptation to a novel target and the extinction of a previously rewarded location. Trials were spaced 15–20 minutes apart, exceeding the range of spatial working memory and distinguishing our paradigm from the “spatial non-matching to place test” of short-term spatial recall. Thus, task learning requires a mouse to constantly refine an association between changing navigational cues and the spatial-contextual rules for maze escape with an increased sensitivity to dentate gyrus function.

**Figure 5:**
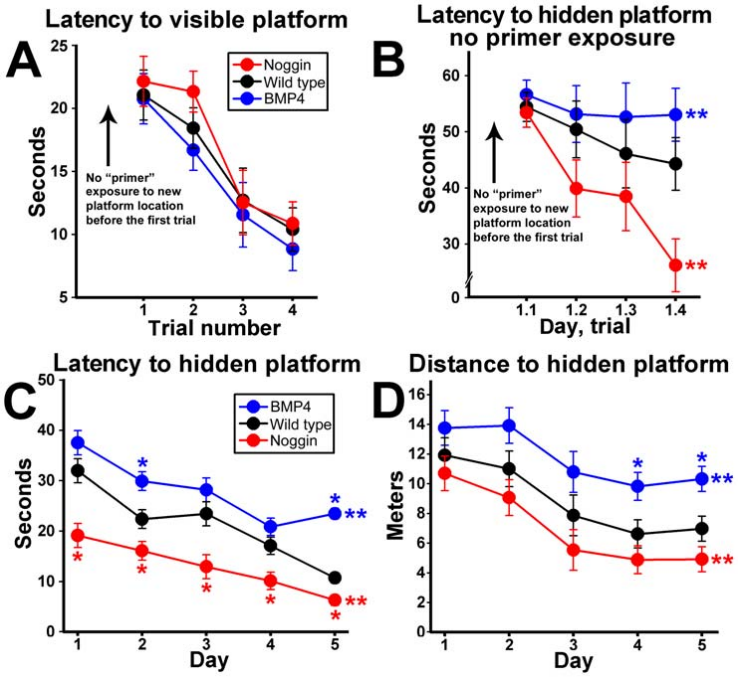
BMP signaling regulates multiple aspects of hippocampus-dependent cognitive performance on the water maze test. Mice were trained on a water maze task requiring daily adjustment to a new target location. (A) All groups performed equally on four trials during a single day when the platform was visible. (B) With a hidden platform, all groups performed equally on the first trial. However thereafter NSE-noggin mice were greatly improved and NSE-BMP4 mice were impaired in the time to find the hidden platform. (C) Daily averages of 4 trials per day for the time required to find a platform hidden each day in a new location. Noggin transgenic mice show superior adaptation to the novel platform location each day between the primer and training trials, and show improved reference learning across the 4 daily trials. BMP4 transgenic mice perform significantly worse than wild type on both measures. (D) The daily average distance swam to reach the platform mirrored the time to reach the platform within each group, indicating equal swimming velocities for all mice. * Differs from wild type at p<0.01 by Bonferroni/Dunn pair-wise comparison. **Significant main effect by multiple ANOVA for group x day comparisons with all groups at p<0.05. The daily average distance swam to reach the platform mirrored the time to reach the platform within each group,

There were no differences in motor abilities of hippocampus-independent learning for the NSE-noggin, wild type and NSE-BMP4 mice on the visible platform test (Fig. 5A). All mice also performed equally on the first trial of a hidden platform test which did not include a preliminary “primer” exposure. Yet thereafter, NSE-noggin mice learned to find the platform faster than WT controls, whereas NSE-BMP4 mice showed no trial-by-trial improvements (Fig. 5B). This controls for differences in initial performance seen in other parts of the experiment due to primer learning (Fig. 5C, Fig. S5A). NSE-noggin mice likewise had better daily scores for the latency and the distance traveled to find the hidden platform (Fig. 5B–D), while NSE-BMP4 mice were impaired relative to wild type. Because the learning curves for the search times and search distances overlap within each strain, the search velocity did not differ between mice (as also seen on the visible platform control test). Slopes of trial-by-trial learning curves for each day indicate that spatial reference learning was also improved in NSE-noggin mice, but impaired in NSE-BMP4 animals relative to controls. On days 1 and 2, search latency decreased by 49% and 45% from trial 1–4 for NSE-noggin mice, compared with 24% and 37% for wild type, and 10% and 2% for NSE-BMP4 mice, irrespective of their initial scores (Fig. S5A). Yet by days 4 and 5, trial-by-trial reference learning was improved in NSE-BMP4 mice despite persistent impairments in adaptive learning on the first trial. Thus, the striking gain or loss of functional ability to refine a spatiotemporal performance construct or to adapt to novel episodic information do not depend on each other in the NSE-noggin or NSE-BMP4 mice. In particular, measures of adaptive learning focused on DG processing functions are also more affected by BMP manipulations (Fig. S5A).

To further assess memory and acquisition, we used an additional maze paradigm which included a 60-second probe trial occurring one hour after the 4^th^ training trial on days 2–5. Mice were scored for the proportion of time spent in the quadrant formerly containing the target, and for the number of times they crossed the former platform location during only the first 15 seconds of the trial. Both measures indicate memory of the target location, yet differences in the two scores can also suggest the refinement of learning behaviors later in the trial. NSE-noggin mice spent 33% of the time in the target quadrant on the very first probe test (Fig. S5B). Wild type mice did not exhibit a memory-based search strategy until day 4, and NSE-BMP4 mice were delayed until the final day of probe testing. For all strains of mice, scores for quadrant search-ratios and for target pass-through events showed similar onset of memory-based search patterns. Yet pass-through scores for NSE-noggin mice remained elevated throughout testing, in contrast to the extinction of full-trial scores for continued searching in the formerly-rewarded quadrant seen on days 4 and 5.

### Transgenic changes in BMP signaling do not affect motor or global learning behaviors

While the tests above are designed to avert confounding factors such as stress, sensorimotor abilities or global skill learning, additional control measurements are needed. We performed a battery of general behavioral tasks including startle/reactivity, rotorod, spontaneous running, activity level and balance beam tests. On the rotorod test for motor ability and learning, all mice showed similar initial performance, indicating equal stamina and coordination. Likewise all mice showed similar learning curves across three additional trials performed at 1-hour intervals, suggesting equivalent motor learning abilities and global response to behavioral training (Fig. S6A). When given access to running wheels, all groups learned to use the wheels equivalently and ran similar distances thereafter (Fig. S6B). This assures the findings in exercise experiments are not explained by differences in the distance run. However studies in C57Bl/6 mice show that running distance varies little between individuals and is not a strong predictor of changes in neurogenesis and learning once above a certain threshold [7]. There were also no differences in coordination and skill learning on a balance beam test (see Methods) measuring initial latencies to cross a beam and return to the home cage floor. On five subsequent trials, all mice displayed equal motor and task learning (Fig. S6C,D). Finally, baseline activity levels and sensorimotor reactivity were equivalent for all mice on control tests for fear conditioning above. This strongly suggests the behavioral phenotypes observed are specific to the functions of the hippocampus.

### Constitutively high levels of BMP signaling block the effects of exercise on hippocampal learning and neurogenesis

**Figure 6:**
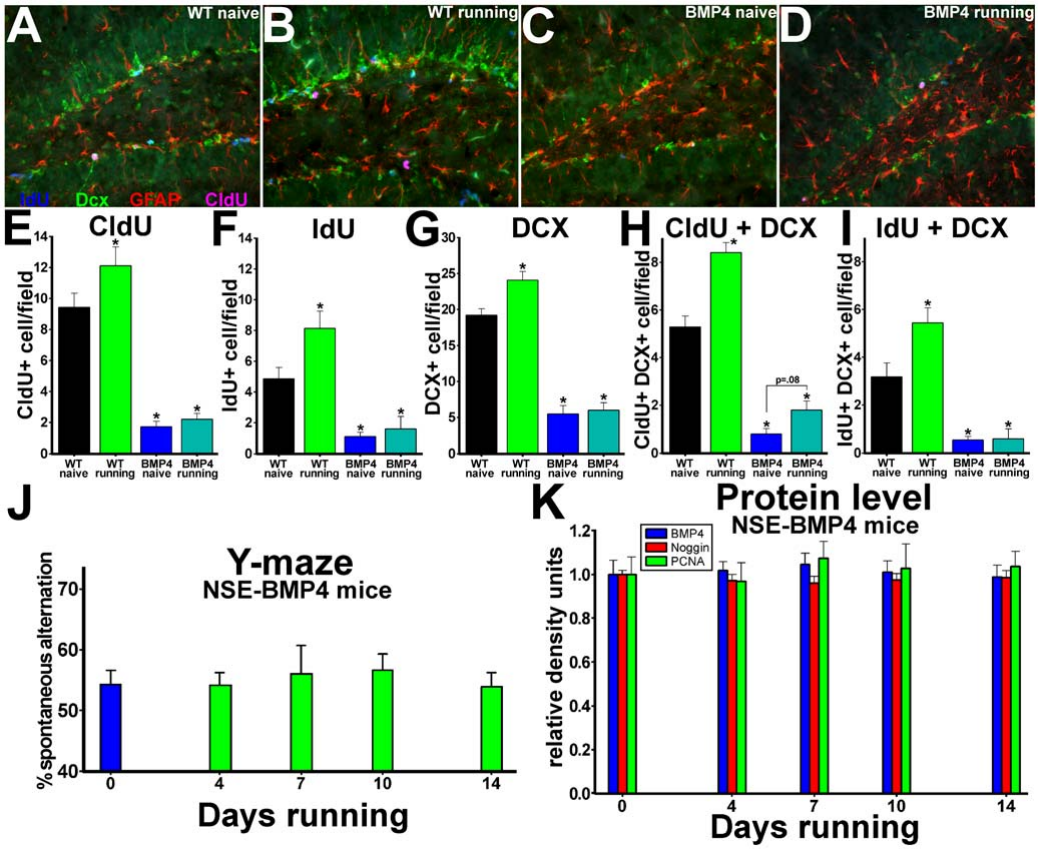
Constitutively high levels of BMP signaling block the effects of exercise. (A–I) Groups of 2 month old NSE-BMP4 mice and age-matched WT controls (N = 16 per group) received CldU for 2 days. Half of each group was exposed to elective exercise for the last 8 days of the 10-day trial, while the other half was maintained in standard housing. On the final 2 days, all animals received IdU. Coronal sections through the dentate gyrus were then immunostained for DCX (green), CldU (pink), IdU (blue), and GFAP (red). Panels A–D show representative merged images for all four labels; (A) Wild type, standard housing; (B) Wild type, running; (C) NSE-BMP4, standard housing; (D) NSE-BMP4, running. (E–I) Unbiased sampling and quantification of cells labeled for (E) CldU; (F) (IdU); (G) DCX; (H)CldU + DCX; (I) IdU + DCX per 40X field. (J–K) Other groups of NSE-BMP4 mice were housed with running wheels for 0, 4, 7, 10 or 14 days and were analyzed for (J) hippocampus-dependent Y-maze performance and (K) hippocampal levels of BMP4, noggin and PCNA proteins. Exercise failed to alter either behavior or levels of any of the proteins in the NSE-BMP4 animals. * Differs from wild type naïve group at p<0.01 by ANOVA and Bonferroni/Dunn pair-wise comparison.

Since the findings above suggest that decreases in BMP signaling might mediate the effects of exercise on hippocampal cognition and neurogenic niche properties, we next tested whether preventing this reduction in BMP activity would block the actions of running. To test directly whether exercise and BMP signaling have opposite effects on SGZ lineage properties, we exposed groups of WT and NSE-BMP4 mice to running wheels or standard housing conditions, bounded by CldU/IdU labeling as described in Figure 2. Since cell labeling with CldU occurred before exercise or naïve treatment, differences in CldU+ cell counts signify the effects of the ensuing treatment on cell survival, differentiation or subsequent division. Because IdU labeling co-terminates with running exposure, differences in co-labeling for IdU and markers of cell identity represent the effects of exercise on cell cycle reentry at specific points along the neural progenitor lineage. In contrast to WT mice, exposure of NSE-BMP4 mice to running did not increase levels of cell proliferation (IdU+), propagation of recently divided cells (CldU+), generation of new neurons (Dcx+), or the stem-like morphology of GFAP+ early progenitors (Fig. 6A–I). Running and naïve NSE-BMP4 mice both had very low levels of doublecortin expression, with no differences in co-labeling for CldU or IdU (Fig. 6G–I). Running exposure in NSE-BMP4 mice also failed to increase the expression of Sox2, nestin, or the co-labeling of Sox2+ cells with CldU or IdU, as seen in WT animals (Fig. S7). Exercise had little additional pro-neurogenic effect in NSE-noggin mice (data not shown), consistent with the saturation of running-induced BMP changes by transgenic over-production. Analysis of cells co-labeled with CldU and NeuN indicates the effects of running on the survival and integration of new cells after their terminal mitosis. Increased BMP signaling reduced the overall number of cells that divided, left the NPC pool, and began to express NeuN 10 days later (CldU+NeuN+). However it did not block the known effects of exercise to promote the survival and neuronal maturation of such cells (Fig. S8). There was no difference between strains or exposures in the span or density of NeuN+ cells across the GCL. Thus, regulation of BMP signaling may focus on the control of NPC division as a key part of the overall effects of exercise on the hippocampus and DG.

To examine the role of reduced BMP signaling as a mechanism for the effects of exercise on hippocampal cognition, we tested the interaction of Y-maze performance, signaling and running duration in NSE-BMP4 mice as described in Figure 1 for wild type animals. Western blot analyses for hippocampal BMP4, noggin and PCNA showed no effect of 0, 4, 7, 10 or 14 days running exposure on levels of signaling proteins and cell proliferation in separate groups (n = 7–8) of NSE-BMP4 mice (Fig. 6K). Running duration also had no effect on Y-maze performance in mice with constitutively high BMP signaling. When subgroups of WT and NSE-BMP4 mice were exposed to running or naïve conditions for the final 8 days of the 10-day paradigm above, improvements in Y-maze, NOR and NLR performance were blocked in the NSE-BMP4 animals (Fig. S9). To determine if the persistence of impairment in NSE-BMP4 mice could be due to the degree of NPC depletion, rather than the loss of an activity-dependent regulatory mechanism, we performed correlation analysis between baseline proliferation levels and cognitive performance within groups of NSE-BMP4 mice. The initial level of NSC reduction is indicated by both the proportion and the number of Sox2+ cells that are co-labeled with CldU. Neither was correlated with test scores after running in NSE-BMP4 mice. Together, our findings support the hypothesis that reduction of BMP signaling is a crucial mechanism in the regulation of SGZ niche properties and hippocampal cognition during exercise exposure. Analysis of other factors such as PSD-95, BDNF and synaptophysin, implicated in exercise-mediated hippocampal signaling and plasticity is provided in Figure S10.

### Cognitive gains in mice with transgenic reduction of BMP signaling require cell proliferation

**Figure 7:**
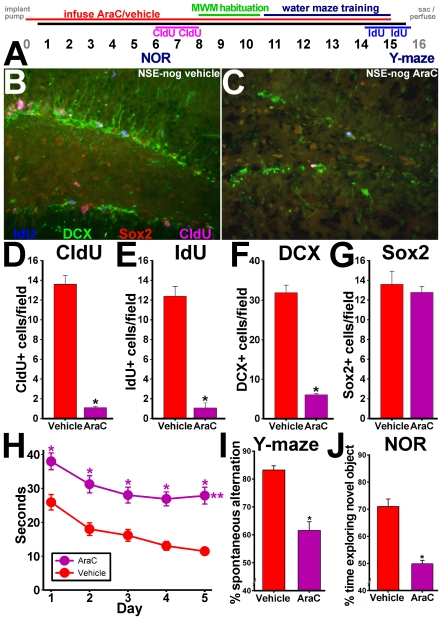
Improved cognitive abilities of NSE-noggin mice require cell proliferation. (A) Two month old NSE-Noggin mice were implanted with an Alzet minipump delivering cytosine arabinoside (AraC) or vehicle intraventricularly (n = 10 per group) for 15 days. A Y-maze test performed on day 3 during a pilot experiment and tests in non-operated mice were used to establish baseline behavior. NOR testing was performed on day 6 and CldU was administered from days 6–8. Thereafter the animals were habituated to the Morris water maze (MWM), and MWM testing was performed on days 11–15. IdU was administered on days 14 and 15, and a Y-maze test was performed on day 15 prior to sacrifice/perfusion of the animals. (B–G) Coronal sections through the dentate gyrus were then immunostained for DCX (green), CldU (pink), IdU (blue), and Sox2 (red). Panels B–C show representative merged images for all four labels. (B) Saline infusion; (C) AraC infusion. (D–G) Unbiased stereological sampling and quantification of cells labeled for (D) CldU; (E) (IdU); (F) DCX; (G) Sox2, per 40X field. (H–J) AraC infusion impaired hippocampus-dependent cognitive abilities of NSE-noggin mice. (H) Latency by day on Morris water maze testing. (I) Y-maze scores. (J) NOR scores. * Differs from vehicle infusion control at p<0.01 by Student's t-test or ANOVA with Bonferroni/Dunn pair-wise comparison. **Significant main effect by multiple ANOVA for group x day comparisons differing from vehicle control at p<0.05.

Since reduction of BMP signaling appears to be sufficient and necessary for the neurogenic and behavioral effects of exercise, we next tested whether the cognitive gains from reduced BMP activity require cell proliferation. To this end, we infused the anti-mitotic drug cytosine arabinoside (AraC) directly into the ventricles of adult mice (see Methods) and measured thymidine analog cell labeling and cognitive behavior. This approach mitigated many of the systemic side effects often seen with peripheral administration of anti-mitotic drugs, along with production of toxic metabolic intermediates by the liver. It also allowed much lower overall dosing, since attaining the target CNS dose doesn't require large systemic doses filtering through the blood-brain barrier. Two groups of P60 male NSE-noggin mice were implanted subdermally with Alzet osmotic pumps connected to indwelling intraventricular cannulae to deliver AraC or vehicle over the course of a 15-day experiment. Pilot studies determined the dosing, diffusion and toxicity of AraC as described in the Methods. On the 2^nd^ through 5^th^ postoperative days, all mice were habituated extensively to the experimental room, handling procedures and equipment. On experimental day 6, all mice were tested for hippocampus-dependent memory on the Novel Object Recognition test, and then received 2 days of treatment with CldU dissolved in drinking water to mark cell division. No other interventions occurred during CldU labeling. After CldU treatment, all mice were habituated and trained on the water maze task as above. On the final two days of testing (experimental days 14–15), mice received IdU in their drinking water to mark cell proliferation at the end of the exposures. After completing water maze training, mice were tested for Y-maze performance and sacrificed for histological analysis. Cannula placement and pump integrity were confirmed in all cases. Figure 7A describes the experimental timeline.

Infusion of AraC significantly reduced the number of CldU- and IdU-labeled cells in the SGZ by 92% and 91%, respectively, and reduced the number of doublecortin immunoreactive cells to only 18% of control levels (Fig. 7A–E) (p<.01). There were only non-significant reductions in the number of cells expressing Sox2 (Fig. 7F) or nestin (Fig. S11), suggesting that AraC infusion has less effect on quiescent NPC subtypes. However, levels of CldU+ or IdU+ cells within individual lineage species, indicating previous or recent cell division, were reduced dramatically by AraC treatment. Cells labeled with CldU and expressing doublecortin or Sox2 were reduced by 98% or 92%, respectively, in AraC-treated mice (Fig. S11A–D) (p<.01). NSE-noggin mice receiving vehicle treatment had levels of cell proliferation and neurogenesis similar to non-operated mice described in Figure 2. No differences were noted in the thickness of the GCL, the shape of other hippocampal structures or the survival of non-dividing cell types.

Mice receiving AraC infusion were significantly impaired on NOR and Y-maze tests performed at the time of CldU and IdU labeling respectively (Fig. 7H,I). AraC infusion also hindered water maze training between these time-points (Fig. 7G). However global functioning, motor abilities and hippocampus-independent learning were not affected by AraC infusion. All mice displayed equal exploration and motivation during NOR habituation and exposure trials and during Y-maze accumulation of arm entries (Fig. S12). Water maze visible platform controls for hippocampus-independent learning and swim speed were also equivalent for all mice tested. On the third day of infusion—before the onset of NPC lineage changes, yet with sufficient time for potential cellular/synaptic effects outside the SGZ—a pilot study found no difference in Y-maze scores (data not shown). Interestingly, AraC produced the greatest deficits in measures focused on the function of the dentate gyrus, such as the NOR and adaptive MWM learning, yet had less effect on spatial reference learning involving other sub-regions (Fig. S12). This suggests the observed learning deficits were not due to acute, systemic, global or pan-hippocampal effects of AraC.

### Intraventricular infusion of noggin reproduces effects of transgenic reduction of BMP signaling on hippocampal neurogenesis and cognitive performance

**Figure 8:**
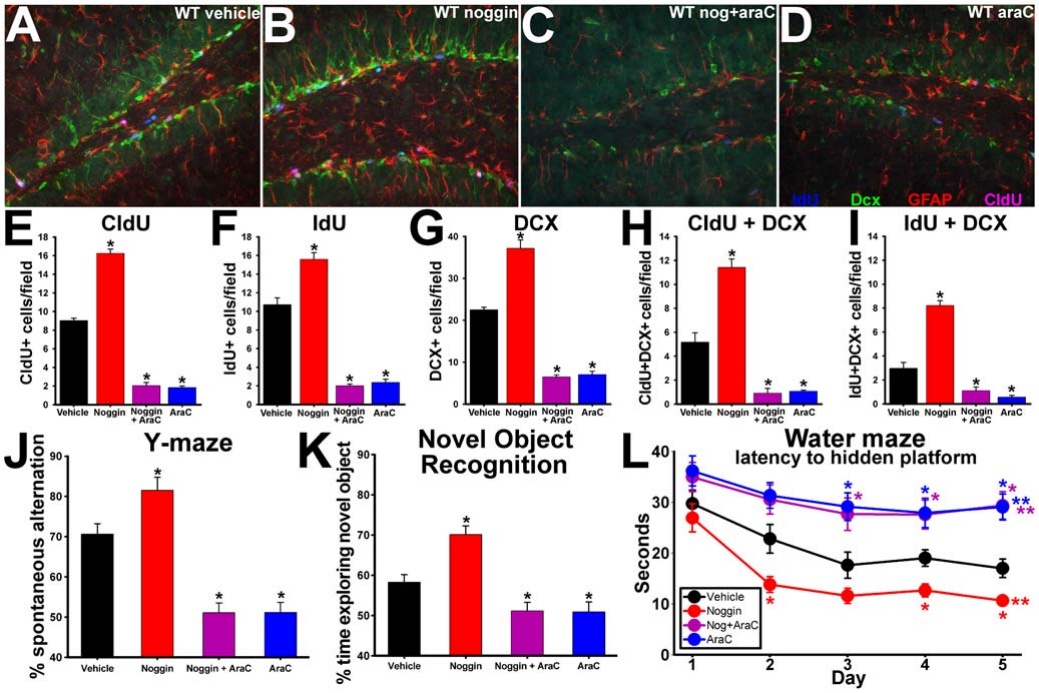
Noggin infusion reproduces effects of exercise and the NSE-noggin phenotype. (A–I) Two month old wild type mice were implanted with Alzet minipumps delivering noggin, AraC, noggin + AraC, or vehicle intraventricularly (n = 10 per group) following the experimental paradigm in Figure 7A. Coronal sections through the dentate gyrus were then immunostained for DCX (green), CldU (pink), IdU (blue), and GFAP (red). Panels A–D show representative merged images for all four labels. (A) Saline; (B) Noggin; (C) Noggin + AraC; (D) AraC (E–H) (E–I) Unbiased sampling and quantification of cells labeled for (E) CldU; (F) (IdU); (G) DCX; (H) CldU + DCX; (I) IdU + DCX. (J–L) Noggin infusion into wild type mice improved scores on hippocampus-dependent behavioral tests in a manner focused on the DG and dependent upon ongoing cell proliferation. (J) Y-maze scores. (K) NOR scores. (L) Latency by day on Morris water maze testing. Analyses of hippocampus-independent behavioral changes and of coronal sections labeled for nestin are provided in Figures S12 and S13. * Differs from saline infusion control at p<0.01 by Bonferroni/Dunn pair-wise comparison. **Significant main effect by multiple ANOVA for group x day comparisons with all groups differing from wild type at p<0.05.

Although the dependence of the learning abilities in the NSE-noggin mice upon cell proliferation suggested a requirement for ongoing niche regulation, this phenotype might be influenced by developmental changes as well. To test whether alterations in BMP signaling dynamically regulate neurogenesis and cognitive function in the normal adult brain, we infused exogenous noggin protein into adult wild type mice in a temporally defined manner (as in the schematic for infusions in Figure 7A). A subset of mice was infused with noggin+AraC to determine whether cognitive effects of noggin would require cell proliferation, as in the NSE-noggin mice above. Histological analysis of mice receiving intraventricular infusion of noggin, AraC, vehicle, or noggin+AraC showed distinct effects of treatment on the proliferative and neurogenic properties of the SGZ niche (Fig. 8A–D). Noggin-infused animals had significantly more cells labeled with CldU (80% increase) or IdU (44% increase) at multiple stages along the NPC lineage (Fig. 8E,F) (p<.01) and markedly increased levels of neurogenesis noted by Dcx expression (68% increase; Fig. 8G) (p<.01) relative to vehicle-infused controls. Mice infused with AraC or noggin+AraC both showed equivalent decreases in cell proliferation in the SGZ niche (76% reduction in CldU and IdU labeling) (p<.01) and decreases in doublecortin expression (73% decrease) (p<.01). However, quiescent nestin+ cell populations were not significantly different between any of the treatment groups (Fig. S13). No changes were noted in general hippocampal morphology or in other cell types outside the neurogenic niche. Thus noggin infusion reproduced the effects of running and transgenic noggin expression on the cellular properties of the SGZ niche.

Prior studies have demonstrated direct changes in BMP signaling in the hippocampus of the NSE-BMP4 and NSE-noggin animals relative to controls [78], [79]. To determine if noggin infusion inhibited BMP signaling in progenitor cells in the hippocampus, we analyzed expression of ID3, a direct target gene of BMP signaling (Fig. S14A–F). Noggin infusion diminished the number of strongly ID3+ cells across the SGZ, and especially in the area of recently divided IdU+ cells, confirming its actions in the SGZ niche. To clarify the hippocampal cell populations affected by noggin-induced ID3 reduction, we analyzed cells co-labeled with ID3 and CldU, IdU or Dcx. Levels of ID3 were significantly decreased in cells adjacent to those expressing IdU and in cells double-positive for IdU and Dcx, consistent with a role promoting cell division along the SGZ lineage (Fig. S14G). ID3 was not reduced in CldU+ cells that had divided 10 days prior to histological analysis; suggesting low ID3 levels denote a cell's capacity for cell cycle reentry, rather than its NPC identity or neuronal differentiation. We also found that noggin infusion appeared to act through endogenous mechanisms linking ID3 and cell cycle events. Noggin only altered the frequency of ID3low/IdU+ cells at the normal sites of cell division, rather than the degree of ID3 reduction or the location of these cells, compared with vehicle treatment. There was little effect of infusion on ID3 expression in cells of the outer third of the GCL or in other hippocampal regions. Further studies will continue to specify the cellular targets of altered BMP signaling, and comparison with hippocampus-specific behavior will provide additional insight.

Since infusion of noggin reproduced the effects of exercise and transgenic over-expression on hippocampal neurogenesis, we next tested for congruent effects on cognitive behavior. Y-maze testing on the third day of infusion, before the presumed onset of SGZ cellular changes, showed no differences between infusion groups. However, noggin infusion produced significant gains in NOR or Y-maze tests performed later during the infusion, at the time of CldU or IdU labeling for increased proliferation (Fig. 8J,K). Infusion of AraC or noggin+AraC impaired performance on these DG-centered tasks relative to vehicle controls. AraC also blocked adaptive learning on a MWM task performed between the times of CldU-IdU labeling, irrespective of co-treatment with noggin. However the loss of cell proliferation did not prevent pan-hippocampal spatial reference learning on the same task (Fig. S12). Noggin infusion without AraC produced remarkable gains in water maze learning (Fig. 8L), reproducing the cognitive phenotype seen in transgenic mice. As in the AraC infusion study, all mice performed equivalently on control tests for hippocampus-independent learning and motor behavior (Fig. S12). Thus, treatment with a single protein was sufficient to reproduce the effects of exercise on adult hippocampal neurogenesis, and to promote hippocampus-dependent cognition on a variety of measures. The intersection of our detailed behavioral analysis with multiple methods to manipulate BMP activity and measure its effects on the NPC lineage supports a novel and crucial role for BMP signaling in the effects of exercise on neurogenesis and cognition.

## Discussion

Exposure to exercise or environmental enrichment is known to regulate the cellular properties of the hippocampal neurogenic niche and to improve hippocampus-dependent cognition. A number of different signaling systems are proposed to influence these changes in hippocampal structure and function [60], [61], [64]–[73], [103]. However an integrated model for the bi-directional regulation of neurogenic potential in response to relevant neural phenomena, which incorporates *in vitro*, *in vivo* and activity-dependent findings, has remained elusive. The temporal relationships between exposure, cell signaling, niche regulation and behavioral changes are also not well characterized. Here we show that BMP signaling plays a central role in mediating the effects of exercise on both neurogenesis and hippocampus-dependent learning and memory. While some studies have addressed the effects of BMP signaling on synaptic strengthening in drosophila [104] and on pre-synaptic plasticity in mice [105], our experiments describe its notable role in the regulation of the hippocampal neurogenic niche. Our findings are also the first to describe the regulation of hippocampal BMP signaling levels in response to activity [106].

BMP signaling reduces neural progenitor cell numbers, proliferation and pro-neuronal fate commitment in the adult SVZ and juvenile SGZ neurogenic niches [78], [79]. Inhibition of BMP signaling by noggin variously has been shown to promote NPC proliferation and differentiation [76], [107] or to increase the numbers of oligodendrocyte precursors [88] in the SVZ. Much less is known about the effects of BMP signaling on hippocampal NPCs at baseline or in relation to activity-dependent phenomena. However the concordance between *in vivo* and *in vitro* findings for BMP-mediated regulation of neurogenic potential in hippocampal NPCs is striking. Cultured SGZ progenitors express both BMP ligands and receptors, suggesting a paracrine signaling mechanism directly within the hippocampus. Inhibition of endogenous BMP activity by noggin is required to uncover the neural stem cell capacity of neurospheres cultured from the SGZ, yet not in those from the SVZ [79]. Culture conditions mimicking neural activity reduce markers of BMP signaling while increasing the size, number and neurogenic potential of neurospheres from the SGZ but not the SVZ. Thus, BMP signaling appears to play a larger dynamic regulatory role in the SGZ than in the SVZ, consistent with the relatively greater effects of exercise therein [79], [108]. These findings describe an *in vitro* model for the local, bi-directional and activity-associated regulation of SGZ progenitor fate that has not been seen with any other pathway to date. Our present experiments test the mechanisms of BMP-mediated regulation of SGZ lineage properties in relation to activity and behavior in a corresponding *in vivo* model system.

Since BMP signaling is known to exist and to regulate niche properties in the adult hippocampus under naïve conditions [79], we first asked if its levels were also influenced by physical activity (Figure 1). We measured levels of BMP signaling via changes noggin and BMP4, which are the main BMP ligand and inhibitor in the hippocampus and are noted functionally by their absolute protein level rather than their sub-cellular location. Levels of BMP4 were reduced and levels of noggin were increased within 4 days of running exposure–well before subsequent changes in cell proliferation by day 7 and in behavior thereafter. qRT-PCR analysis mirrored changes in noggin and BMP4 proteins, and showed no compensatory changes in other BMP-related transcripts which cumulatively might mitigate our findings (data not shown). Immunostaining for pSMAD1/5/8 confirmed the cells and locations responding to BMP changes in the DG of WT mice exposed to exercise for 8 days relative to naïve controls. Together these findings show that physical activity regulates hippocampal BMP signaling, and suggest that BMP alterations could underlie changes in hippocampal behavior or the cellular properties of the hippocampus during exercise. We therefore asked if manipulation of BMP signaling separately from its regulation by exposure to environmental stimuli could recapitulate the phenotypes produced by exercise.

To clarify the stages at which exercise and BMP signaling act on the SGZ niche, we developed a “dual time-point, dual lineage marker” paradigm for cell fate and lineage analysis. This allows us to analyze patterns of NPC subtype regulation in relation to changes in both a mature neuronal marker and behavioral outcomes. We found that exercise and inhibition of BMP signaling increased the incorporation of both CldU (given early during the experiment) and IdU (given toward the end) in multiple progenitor subtypes. Incorporation of IdU was increased in both early stem/progenitor cells, and in later precursors and neuroblasts. This indicates that exercise and noggin increased proliferation across the SGZ lineage: from quiescent multipotent progenitors (Sox2, GFAP or nestin+ cells), to Dcx+ species ontologically closer to functional integration. In fact, Dcx+ progenitors already receive medial perforant path (MPP) input and project to interneurons linking the DG and CA3, even before the time of their terminal mitosis [109]. Within 3–4 days of their final division, such cells begin to form their own mossy fiber synaptic connections with CA3 pyramidal neurons [42], [109], [110]. Individually, the contribution of new cells to information processing within the hippocampal circuit will follow a continuum from tonic to synaptic, GABAergic to glutamatergic, transmission [109], [111]–[114]. However the distinct properties of young and old cells effectively splits DG granule cell populations into functional subgroups–the balance of which can be regulated on a time scale much faster than for the progression of an individual cell through the entire SGZ lineage [17]–[19], [21], [25], [26]. Our findings, and other studies showing cellular and behavioral gains after 5–7 days [7], [71], [115]–[117], support a functional role for the dynamic balance of GC populations.

Inhibition of BMP signaling by infusion or transgenic overexpression of noggin significantly enhanced hippocampus-dependent cognition–even in excess of gains seen from exposure to exercise or environmental enrichment. Remarkably, manipulation of this single pathway can either enhance or impair hippocampal cognitive functions and modulate the effects of exercise. Noggin infusion into adult wild type mice produced the same changes seen in the NSE-noggin animals, indicating that the phenotype of the transgenic mice does not stem from developmental changes. Noggin infusion also reproduced the temporal, histological and behavioral outcomes seen from exercise exposure. The bivalent behavioral phenotypes of the NSE-noggin and NSE-BMP4 mice were remarkably strong and consistent across an array of tests for hippocampus-dependent learning. While these findings differ from the mixed outcomes seen in prior studies, our experimental model focuses on the function and regulation of cell lineages in the DG, rather than the level of cell division required for continued spatiotemporal learning. However, changes in the levels of neurogenesis in our experiments are at least comparable with other reports [51]–[59], [61]–[63]. Our behavioral tests entailed enhanced ethological sensitivity, habituation periods and internal controls for confounding factors. No differences were seen between groups of mice for non-specific motor behavior, activity level, arousal or attention in any of the experiments.

It is especially notable that so many aspects of hippocampus-dependent cognition were enhanced in the noggin-infused and transgenic mice, while other hippocampus-independent behaviors were unaffected. Performance on the Y-maze involves a form of hippocampal spatial memory that does not require task-based rule learning or recall. The dependence on distal cues to discriminate which identical arm is occupied and which should be chosen next in the sequence suggests DG-mediated processing functions. Fear conditioning and NOR/NLR tests utilize separate encoding and recall exposures to measure learning and memory across a span of time. The modified water maze paradigm is designed to test both the recollection of the platform location and the overall task rules, as well as the integration of novel episodic information within this paradigm. Thus, successful maze performance requires synchronized encoding, retrieval and discrimination of relevant spatial information. This behavioral procedure recapitulates the information processing function of simultaneous pattern separation and pattern completion by the DG-CA3 circuit. Likewise, the ability to distinguish novel spatial features on the NLR test may highlight the role of the DG in spatial coincidence detection [46], [47], [102]. Discrimination of object features on the NOR test is also proposed to be an extension of DG-centered information coding, with additional processing by CA3 and CA1 [46], [48], [95]. Interestingly, on measures of spatial reference learning and probe trial search strategies, which are less dependent on the presumed functions of the DG, NSE-BMP4 mice displayed intact but delayed learning abilities (Fig. 5 and S5). Likewise, AraC infusion had little affect on daily trial-by-trial MWM scores for spatial reference learning (Fig. S12), but caused notable impairment in tasks focused on DG processing functions. The findings that exogenous or transgenic manipulation of BMP signaling and niche properties produced such strong, bidirectional effects on tests requiring discrimination and integration of novel information suggest that BMP signaling plays a central role in hippocampus-dependent cognition.

We next sought to determine whether the down-regulation of BMP signaling in the hippocampus is required for enhancement of hippocampal cognition and neurogenesis by exercise. Consistent with our hypothesis, running did not increase levels of SGZ cells expressing Sox2, nestin or Dcx in NSE-BMP4 mice. Nor did exercise promote cell division within each NPC subtype in mice over-expressing BMP4, as seen in WT controls. While NSE-BMP4 mice had reduced baseline proliferation levels, aged mice and other strains with similarly low initial levels of cell division still displayed robust gains in neurogenesis after exercise [10], [92]. Thus, NSE-BMP4 mice seem to lack an activity-dependent regulatory mechanism for cell cycle reentry, which persists in other animals irrespective of reductions in the substrate on which it acts. Further, baseline NPC counts were not correlated with cognitive test scores after running in individual NSE-BMP4 mice. This suggests that over-expression of BMP4 does not simply deplete a source used for other activity-dependent phenomena, but opposes a mechanism of regulation of SGZ lineage properties and functions during exercise. In fact, the effect of exercise to promote the survival and maturation of cells exiting the NPC pool and becoming NeuN+ neurons was still present in these animals. Likewise, NSE-noggin mice, which might have greater cellular stores for other mechanisms but a relative saturation of the BMP modulation seen in Figure 1, showed little additional benefit from running exposure. Constitutively high levels of BMP signaling also blocked improvements from exercise on Y-maze, NOR and NLR performance. However, the ability to regulate other activity-mediated signaling pathways was maintained, since we found that levels of BDNF were increased equally in NSE-BMP4 and wild type mice after exercise (Fig. S10). NSE-BMP4 mice also retained hippocampal functions less dependent on the DG. These results indicate that regulation of BMP signaling is both sufficient and necessary for the effects of exercise on hippocampal cognition and the cellular properties of the SGZ niche.

Other factors are also known to increase in the brain or in the general circulation during exercise and to influence levels of SGZ proliferation. VEGF and IGF-1 are produced largely outside the brain, yet may be involved in the regulation of hippocampal function by activity. Within the hippocampus, levels of mRNA for VEGF, IFG-1 or BDNF may be up-regulated during running, yet such findings have been inconsistent [70]–[72], [116]–[119]. The time course for these changes in relation to the onset of improved learning and other hippocampal outcomes likewise has not been determined in detail [67], [71]–[73], [116]–[119]. Treatment of cultured hippocampal NPCs with BDNF promotes survival and neurogenesis [120], and VEGF or IGF-1 can support proliferation or survival in low-mitogen conditions [72], [121]. However other studies suggest a downstream role for BMP signaling in these effects [122]. Infusion of VEGF, BDNF or IGF-1 into the brain can support neurogenesis in the SVZ and the SGZ [123]–[125], but does not reliably reproduce the cognitive benefits of exercise. Conversely, reduction of BDNF signaling by genetic knockdown [65], [66], [126], or of VEGF or IGF-1 signaling by peripheral administration of blocking antibodies [67], [72], [73], reduces the normal actions of running on learning and neurogenesis. However, only BDNF knockdown consistently causes impairments in naïve mice. This suggests that BDNF may help to regulate levels of SGZ neurogenesis at baseline and during activity, but that IGF-1 and VEGF may prompt neurogenesis and learning only during exercise. Indeed, peripheral administration of blocking antibodies to VEGF and IGF-1 has no effect on these outcomes in naïve mice. However BDNF, VEGF, and IGF-1 all have notable extra-hippocampal effects on synaptic plasticity, stabilization and release probability, and on cellular metabolism [127]–[132]. There is also much less evidence for the bi-directional regulation of cell cycle reentry and fate commitment by these factors than there is for their promotion of NPC survival later in the neuronal lineage. BMP signaling levels are regulated bi-directionally as part of both normal homeostasis and activity-dependent processes; and manipulation of these levels yields similar bi-directional effects on cell fate decisions and cognitive function in the hippocampus.

The functional relationship between neurogenesis and behavior remains controversial [29]–[34], [51]–[56], and is not the focus of our experiments. Yet we found that intraventricular infusion of AraC virtually abolished the beneficial effects of reduced BMP signaling on hippocampus-dependent cognition. This suggests that the behavioral effects of noggin require mitotic progenitor cell populations in the neurogenic niche. Infusion directly into the ventricles improves the specificity of administration, and reduces concerns of systemic side effects which might confound experimental outcomes. Indeed, control tests did not indicate any changes in general well-being, motivation, motor performance or hippocampus-independent behavior between AraC- or vehicle-infused mice. Nor were there any general changes in other hippocampal structures, the thickness of the dentate granule cell layer above the SGZ, or the survival of previously divided (CldU+) cell populations. AraC likewise did not affect the numbers of cells expressing Sox2 or nestin, consistent with their relative quiescence. Thus, cognitive performance is associated more with cell progression through the neurogenic lineage and the number of new neurons than with the number of stem cells in the DG.

Infusion of noggin or noggin+AraC both reduced the expression of the BMP target gene ID3 in the SGZ, but only noggin increased hippocampal neurogenesis and cognitive performance. In fact, infusion with noggin+AraC impaired learning equivalently to AraC infusion alone. This suggests that the behavioral benefits of reduced BMP signaling require ongoing cell proliferation in the adult brain. Noggin infusion reduced expression of BMP target genes directly in the SGZ, and improved hippocampus-dependent behaviors seen to require proliferative capabilities unique to the SGZ and SVZ. However actions outside the hippocampus can not be ruled out. To clarify the mechanism for the effects of noggin on neural function, we analyzed the point of action of BMP reduction within the DG using a novel system to measure temporal/lineage progression and cell signaling markers all within the same section. ID3 expression was reduced in and near cells labeled with IdU marking recent division, and even in later lineage species expressing Dcx at the time of their final divisions. However other Dcx+ cells migrating out of the SGZ niche, and/or co-labeled with CldU marking past division, expressed increased levels of ID3. This suggests that reduced BMP signaling and ID3 expression are associated with localized increases in neurogenic potential and with cell cycle reentry of multiple NPC species along the SGZ lineage. It does not seem to designate a broad progenitor identity, or the survival and neuronal maturation of post-mitotic cells during neurogenesis. The morphology of GFAP+ progenitor cells is another important indicator of BMP signaling levels and NSC potential [78]. Noggin infusion promoted a radial, bipolar GFAP+ cell type denoting reduced BMP effects in SGZ progenitors, as was also noted by ID3 expression levels. Yet only in the absence of AraC were these changes associated with increased proliferation, neurogenesis and hippocampal cognition. Further experiments will test the location and cell types responsible for the effects of BMP inhibition more directly.

The intersection of these findings with the results from the transgenic and exercise experiments above describes a central role for BMP signaling in the regulation of hippocampal cognition and the properties of the neurogenic niche. In particular, inhibition of BMP signaling appears to be both sufficient and necessary for the cellular and behavioral effects of exercise exposure focused on mitotic NPC populations and the information coding functions of the dentate gyrus. While no single result proves a direct causal relationship between exposure, time, signaling mechanism, location, cell type and function; our findings address multiple combinations of these factors in great detail. Their remarkable confluence and the striking cellular and behavioral phenotypes of the transgenic and noggin-infused animals all point to a novel and crucial role for BMP signaling in the cellular properties, function and physiological regulation of the adult hippocampal neurogenic niche.

## Methods

### Animals

The creation of the NSE-noggin and NSE-BMP4 transgenic mice was described previously [78], [89], [90]. Mice were initially derived on the FVB background strain and were backcrossed 5–7 generations onto C57Bl/6. All mice were 60–75 days old during experiments, and were housed in groups of 3–5 mice per cage with food and water *ad libitum*. All animals were males, except in the experiment measuring changes in BMP signaling after exposure to exercise, which used equal numbers of male and female mice. No differences were noted by gender, allowing data to be pooled. Mice were housed in a facility with a 14/10 hour light/dark cycle. Experimental protocols were approved by IACUC and Northwestern University's CCM. Behavioral experiments were designed with special ethological sensitivity to the mouse's perspective and sensory experience as a nocturnal, terrestrial prey animal.

### Running exposure

P60 C57Bl/6 mice were transferred in groups of 3–4 to cages containing a standard wire exercise wheel suspended from the cage top and a Fast-Trac running disc (Bio-Serv). Mice were continuously housed with elective exposure to exercise for a given duration and were observed and randomly sampled for differences in running duration, distance and acquisition on a cage-by-cage and individual-by-individual basis to test equality between groups.

### Western blot analysis

Different groups of C57Bl/6 or NSE-BMP4 mice were housed 3–4 per cage with free access to a running wheel and disc as described above for 0(naïve), 4, 7, 10 or 14 days (n = 5–7 per group). After exposure, mice were sacrificed by CO_2_ narcosis and their brains were rapidly removed into ice-cold PBS with HALT protease inhibitors (Pierce). Both hippocampi were dissected out with care to exclude the surrounding white matter and any portion of the adjoining lateral ventricle. Hippocampi from each mouse were collected in tubes containing 150 µl ice cold T-PER Tissue Protein Extraction Reagent (Pierce) with 1X HALT protease inhibitors (Pierce) and were manually homogenized on ice with a plastic tissue pestle. The samples of cell lysate were then centrifuged at 4°C at 2000 rpm for 10 min followed by 4000 rpm for 5 min and 6000 rpm for 1 min, and the supernatant was collected into a new tube on ice. Total protein concentration was quantified using a BCA Protein Assay Kit (Pierce) and spetrophotometry. Protein samples were mixed with an equal volume of denaturing buffer and were boiled for 20 min. 55–65 µg of total protein from each sample was then loaded onto a 4–20% Tris-HCl Ready Gel (Bio-Rad) such that each lane signified a unique animal. After electrophoresis and transfer to a Trans-Blot nitrocellulose membrane (Bio-Rad), membranes were washed in 1X TBST and then blocked in 5% non-fat dry milk in TBST. After blocking, membranes were washed 3X and then incubated with antibodies for noggin (rabbit, 1∶3850; Chemicon), BMP4 (mouse, 1∶5000; Chemicon), PCNA (mouse, 1∶5000, BD), BDNF (rabbit, 1∶1000, Santa Cruz), synaptophysin (mouse, 1∶5000, Chemicon), PSD-95 (mouse, 1∶25,000; NeuroMab), and actin (goat, 1∶1000; Santa Cruz). Blots were then washed 3x, primary antibodies were labeled with appropriate HRP-conjugated secondary antibodies (Santa Cruz), washed 3x, developed with Luminol chemiluminescent reagent (Santa Cruz) and exposed to photographic film. Films were scanned into a computer and analyzed by densitometry using Image J software. Measurements for band densitometry were normalized to actin loading controls for each sample. Statistical analysis was by ANOVA with Bonferroni/Dunn post-hoc comparisons for the effects of running duration on protein densitometry.

### qRT-PCR

Mice were housed with exposure to running wheels, as above, for 0 (naïve controls), 2, 4, 7, 10 or 14 days (n = 7–10 per group). At the end of the exercise exposure, mice were tested for spontaneous alternation exploratory patterns on a radial Y-maze test of hippocampus-dependent spatial-sequential processing. Since this test consists of a single discreet exposure and involves no cognitive training, it is unlikely to have an impact on the other experimental measures. After testing, mice were euthanized and their hippocampi were collected as above into ice cold PBS under RNase-free conditions. RNA was prepared from whole hippocampi using an RNAqueous-4PCR kit (Ambion) and was reverse-transcribed to cDNA using the ThermoScript RT PCR System (Invitrogen). Quantitative PCR was performed using the Applied Biosystems ABI Prism 7700 Sequence Detection System, and measurements of SYBR Green (Applied Biosystems) fluorescent dye incorporation were used to calculate the critical threshold (CT) cycle number for noggin, BMP4 and GAPDH mRNA. A pilot study also measured transcript levels of BMP2, BMP7, BMP10, neurognesin and chordin. Primer sequences are available upon request. CT values were normalized to GAPDH for each animal. CT differences between naïve and 2, 4, 7 or 10 day running groups were converted to log 2 values for fold change in mRNA and analyzed by ANOVA with Bonferroni/Dunn post-hoc comparisons.

### CldU and IdU

Injections occurred over the course of 10 days. On the first two days, mice received 4 daily i.p. injections of CldU (Sigma) dissolved 10 mg/ml in sterile saline every 2.5 hours. On the last two days, mice received IdU (Sigma) using equivalent methods and molar dosing as with CldU. Mice were sacrificed for immunohistochemistry on the day following their last injection. CldU and IdU dosing was adjusted volumetrically to the molar equivalent of 50 mg/kg BrdU for each animal (42.5 mg/kg CldU and 57.5 mg/kg IdU). For the infusion studies, both thymidine analogs were provided via drinking water treatment for at the molar equivalent of 1 mg/ml BrdU dissolved in tap water with 2.5% sucrose for 2 days. In pilot and previous studies [79], [133], [134], this route gave equal dosing and labeling as the injection paradigm described.

### Immunohistochemistry

On the day following the last injection, mice (n = 4–5 per group) were perfused with ice-cold buffered saline solution, then with 4% paraformaldehyde (PFA). Brains were removed, post-fixed in 4% PFA, dehydrated in 30% sucrose in PBS, and rapidly frozen for sectioning on a Leica CM3050S cryostat. 10 µm coronal sections were mounted serially, 10 per slide, with successive slides representing the rostro-caudal progression through the hippocampus. Three slides were selected from each animal, balanced along the a-p axis within and between subjects, and the first and last sections were counted from each slide. This defines an objective stereological sampling method to quantify cellular data for each subject and account for internal trends and variability. Sections were processed for antigen retrieval by incubating in 10 mM sodium citrate pH 7.1 at 95°C for 20 min. After cooling for 30–40 min, sections were washed in PBS, and were incubated in 2 N HCl at 37°C for 20 min and cooled to room temp for 10 min to expose epitopes in the DNA. After acid treatment, sections were neutralized in 0.1 M Borax pH 8.5 for 5 min, washed in PBS, blocked in 10% fetal bovine serum for 2 hours, washed again, and exposed to primary antibodies diluted in PBS with 1% BSA and 0.25% Triton X-100 overnight at 4°C. Primary antibodies included CldU (rat anti-BrdU, 1∶250 Accurate); IdU (mouse anti-BrdU, 1∶250 Becton Dickinson); Sox2 (rabbit 1∶200 Chemicon); nestin (rabbit 1∶400 Abcam); GFAP (rabbit 1∶400 Dako); ID3 (rabbit 1∶100 Santa Cruz); NeuN (mouse 1∶500 Chemicon); pSMAD1/5/8 (rabbit 1∶100 Cell Signaling); and doublecortin (goat 1∶400 Santa Cruz). Sections were then washed in PBS and incubated for 2 hours with appropriate fluorescent-labeled secondary antibodies (Alexa-Fluor-405/488/594/647; Molecular Probes) to visualize the primary antibodies. Finally, sections were washed, counterstained with Hoechst 33342 (Sigma), and mounted with cover slips using ProLong Gold reagent (Invitrogen). Unbiased stereological sampling of 2 sections from each of 3 slides per subject assured balanced and objective counts. The same 40X frame was selected for each section and the number of cells per field was counted using pre-defined, morphological and ultra-structural criteria. Data points from each field were used to determine the mean and variability of cell counts for each subject. We did not apply a conversion factor to extrapolate this data into counts per hippocampus, since normalized findings lose accuracy and validity in describing observed changes and variance in the outcome measures. Yet, readers preferring this convention may multiply the reported values by 500 (2 significant figures). Mean cell counts throughout the hippocampus from each animal were used as a single data point for one-way ANOVA (after tests for homogeneity of the set: *cells x section x slide x animal*). This strategy assures the normality of the data, and provides a more conservative analysis than ANOVA with repeated measures. Levels of pSMAD1/5/8 or ID3 expression were analyzed by densitometry of objectively selected regions using Image J software.

### Morris water maze: apparatus and habituation

The water maze apparatus consisted of a circular pool 120 cm in diameter, a 10×10 cm escape platform, an array of extra-maze visual navigational cues hung from curtains surrounding the pool area and an overhead camera connected to a computer running video tracking software. Water within the pool was kept at 24–25°C and was made opaque with non-toxic white tempura paint. During the week before maze training, mice were handled extensively and were habituated to the training room. Mice were also habituated to the pool components by placing their cage on the platform just above the waterline and allowing them to observe the testing arena while still warm and dry. On the third day before testing, mice were incrementally habituated to the pool and platform, which stood 1 cm above the waterline. Mice were taught to rest on the platform in this new environment, and to climb onto the platform from the water and into the experimenter's hand for return to their home cage. On the second day before training, the platform was submerged 0.75 cm below the waterline, but was marked with a 4×4×4 cm metal cube. Mice learned to rest on the submerged platform and the cube, and that they would be returned to their home cage after remaining on the platform for 10 sec. After 5 of these exposures, mice were allowed to rest for two hours and were then tested on a visually cued version of the water maze test (visible platform test). The submerged platform and visible cube marker were moved to a new location within the pool, and mice were tested for their ability to reach the platform from different starting points. Four search trials began from different starting positions, named N, E, S, W, in pseudo-random order for each mouse. The time to find the visually cued platform and the path traveled was recorded for each trial. On the final day before training, mice were placed on the submerged platform with the visible cue present. After 5 seconds, mice were nudged off the platform and allowed to climb back on. After an additional 5 seconds, the cue was removed, and mice were nudged into the water and immediately guided back to the platform where they were allowed to rest for 10 seconds before returning to their home cage. During 3–4 following exposures, mice were placed directly onto the unmarked submerged platform for 5 seconds, were nudged off, and were allowed to swim until they regained the platform and rested for 10 seconds without guidance.

### Morris water maze: training procedure

After the three days of habituation, NSE-BMP4, wild type and NSE-noggin mice (n = 10, 15, 10) began a 5-day course of training on a submerged platform version of the water maze. Each day, the target platform location was moved to a different quadrant within the pool, with the location on day 5 repeating that of day 1. Two hours before testing, mice were placed on the platform in its new location for 15 seconds as a “primer” exposure for the 4 subsequent training trials that day. To begin each trial, mice were placed in the water facing the wall at the N, E, S, or W starting locations, with the sequence arranged in pseudo-random order to avoid quadrant bias within groups. The 4 trials were spaced 15–20 minutes apart: a span well outside the range of working memory for mice, and requiring the hippocampus for recall and learning. Each trial ended when the mouse climbed upon the target platform or after 60 seconds of search time. At the end of each trial (mice not finding the platform were guided to it), mice were allowed to rest on the platform for 10 seconds, reinforcing the associative pairing of location-reward. Mice were then dried off, returned to their home cage and monitored for recovery of warmth. Each trial was stopped with a pneumatic input device connected to a computer running Maze 2020 and HVS Image software. All trials were also recorded with HVS Image video tracking equipment, which measured parameters including path length, average speed and latency to platform. An additional experiment was conducted to control for the effect of initial performance on daily learning curves, and to measure acquisition of learning criteria using a series of “probe trial” tests. NSE-noggin, wild type, and NSE-BMP4 mice (n = 11, 10, 11) received 3 days of habituation and visible platform testing, as in the primary experiment above, but were not exposed to the new platform location prior to the first trial on training day 1. After learning the task parameters on the first day, days 2–5 contained an extra trial 60 minutes after the 4^th^ training trial in which the hidden platform was removed from the pool. Mice searched throughout the pool for 60 seconds. The proportion of search-time spent in the former target quadrant and the number of “pass through” events over the former platform location during the first 15 seconds were recorded. All statistical analysis was by 2 or 3-way ANOVA for main effects of genotype, trial, or day on search latency/distance, followed by Bonferroni/Dunn post-hoc tests for multiple pair-wise comparisons.

### Spontaneous alternation Y-maze

This is a hippocampus-dependent spatial working memory task which does not require any training, habituation of reinforcement. The task is designed to measure the efficiency of a mouse's exploration strategy in a new environment. The Y-maze consists of three radial arms measuring 60 cm in length, 18 cm deep, 4 cm wide at the bottom and 14 cm wide at the top, constructed of translucent acrylic, providing no distinguishing marks within the maze but allowing a view of the surrounding room. Thus, maze navigation requires hippocampus-dependent spatial-sequential guidance from extra-maze visual cues. Mice were placed into the end of one arm, and were allowed to explore the maze freely for 5 minutes. The sequence and total number of arms entered was recorded for each mouse. Arm entries were counted when a mouse's hind paws had completely left the central hub, and the mouse had taken two steps down the chosen arm. Re-entries into the same arm without completely leaving the central hub were not scored. The efficiency of exploration was measured counting the number of entry triads (any 3 consecutive arm entries) which contain entries into all three arms, divided by the total number of entry triads (total number of arms entered, minus 2). This number indicates the percentage of non-repeat arm entries, and ranges from 100% for perfect alternation to 50% for chance levels of performance. The outcome measure, % alternation, is calculated as follows: *% alternation = [(triads with 3 unique arm entries)/(total entries−2)]×100*. To control for masking of true alternation by an “always turn right/left” arm choice strategy, a pilot study measured perseveration by returning the mouse to the starting position after making the first arm choice. On the following arm choice, choosing the third un-entered arm indicated an alternation-based exploration strategy. Statistical analysis was by ANOVA with Bonferroni/Dunn post-hoc tests for pair-wise comparisons.

### Novel object recognition and novel location recognition tests

Mice were habituated to the testing arena, consisting of an open, white 35×50×25 cm plastic tub for a 5 min exposure 3 times a day for two days before testing. On the day of testing, mice were placed in the open arena and allowed to explore for 1 minute. Mice were returned to their home cage, the arena was cleaned, and two identical objects were placed into the field in bilateral, mirror image orientation to the mouse's starting position at the front and center of the field. A pilot study was conducted to find a set of objects which appeared to be non-threatening and equally interesting to mice. Upon re-introduction to the field with the two identical objects, mice were allowed to investigate the arena for a cumulative 30 seconds of object-exploration time. Exploration was defined as active examination in which the mouse was sniffing, touching or scrutinizing an object, and not merely sitting in its vicinity or rearing up against it. Cumulative exploration time for each object, and the number and duration of individual exploring events, were recorded using Stopwatch Plus software. After this exposure trial, mice were returned to their home cage for a given delay period, and the arena and objects were cleaned with 70% EtOH to remove all olfactory cues. For the novel object recognition test, one of the familiar objects was replaced with a novel object, and mice were re-introduced to the arena after 1.5, 3 or 14 hours, with no mouse tested twice. For the novel location recognition test, one of the familiar objects was moved to a new location within the arena, and mice were re-introduced for the memory trial after 2.5 hours. During the recall trial, mice were again allowed to explore the arena for a cumulative 30 seconds of object-exploration time, and each exploratory event was recorded. Mice have a natural predilection for new items in their environment, so the percentage of time at the new object or location indicates the mouse's degree of memory for the familiar object or location. Statistical analysis was by 2-way ANOVA with Bonferroni/Dunn post-hoc comparisons for the effects of genotype on object/location recognition for multiple delay time points.

### Fear conditioning

Male, 60–70 day old NSE-noggin, wild type and NSE-BMP4 (n = 9–10 per group) were tested using a computer-controlled activity monitor and tone/shock generator (TSE, Bad Homberg, Germany). On the first day of testing, mice were exposed to a Plexiglas chamber (35×20×20 cm with a wire shock grid floor (stainless steel bars, 3 mm diameter, spaced 9 mm apart) for 3 minutes. This chamber was contained inside a second, sound-attenuated chamber (58×30×27 cm) with a gray interior, a 12-W light source and a speaker (Conrad, KT-25-DT). At the end of the 3 minute simple exposure, mice were played a 75 dB, 10 kHz tone for 30 seconds, during the last 2 seconds of which they received a 0.7 mA constant current foot shock. During these exposures to the chamber, tone and shock, the activity level (cm/s) for each mouse was recorded by an infrared beam array detector. After 24 hours, mice were tested for contextual and cued conditioning to the chamber and tone. For contextual conditioning, mice were re-exposed to the training chamber for 3 minutes without tone or shock, and were recorded for stereotyped freezing behavior and general activity level. Afterwards, mice were re-assessed for baseline activity levels, and then tested for cued conditioning to the tone stimulus in a novel context. This environment resembled the training chamber, but had a20-W light source, a solid white floor, and was cleaned with 1% acetic acid. General activity levels were measured for 60 seconds; then mice were exposed to the 10 kHz tone for 3 minutes and scored for freezing to the cued stimulus. As with the measurements for freezing to contextual stimuli, behavior was scored by sampling every 10 seconds, for a total of 18 measurements. Statistical analysis was by 1-way ANOVA with Bonferroni/Dunn post-hoc tests for the effects of genotype on freezing behavior.

### Rotorod test

Mice (n = 9–10 per group) received 4 trial of testing on a rotorod device (Med Associates), beginning at 4 rpm and accelerating to 40 rpm over 4 minutes. Trials were spaced 60 minutes apart and were terminated when the mouse fell or completed two revolutions about the rod without regaining locomotion. Statistical analysis was by 2-way ANOVA with Bonferroni/Dunn post-hoc comparisons for the effects of genotype on initial scores and learning.

### Balance beam test

NSE-noggin, wild type and NSE-BMP4 mice (n = 9 per group) were tested for motor coordination and task learning on a balance beam test using a 80×1 cm dowel extending from across the top of the home cage, out above the opening of a 60×60×60 cm padded cardboard box. Mice were placed on the dowel, 20 cm from its end, 40 cm from the edge of the home cage and were scored for falls, latency to return to the cage opening and latency to return to the cage floor. Each trial lasted until the mouse fell or successfully returned to the home cage. Falls and failures to climb down to the cage floor were both scored as a latency of 90 seconds, yet both events were rare. Mice were tested for 6 trials, spaced 60 minutes apart. Statistical analysis was by 2-way ANOVA with Bonferroni/Dunn post hoc comparisons for the effects of genotype and genotype*trial on coordination and task learning (for beam crossing and descent).

### Infusion

Adult male mice were anesthetized in isoflurane and incised to reveal the cranium from lambda to the occipito-cranial junction. Using blunt dissection, a subcutaneous pocket to receive the implanted osmotic pump was made from the caudal aspect of the cranial incision toward the tail. Using sterotactic guidance, a 1 mm cranial defect was drilled −0.4 mm and 1 mm lateral to bregma. The dura was opened using a 25-guage needle and the Alzet brain infusion cannula was placed within the lateral ventricle. An Alzet micro-osmotic pump (#1002), pre-loaded with the factor/drug, and attached to the cannula with a 1 cm plastic catheter was implanted into the subcutaneous pocket prior to insertion of the cannula into the ventricle. Each pump delivered noggin (R&D) at 50 ng/µl or AraC (2% Sigma) for 14 days at a rate of 0.25 µl/hr. Mice show full behavioral and functional recovery very soon after surgery. Infusion was performed in conjunction with cell labeling and cognitive testing as described above. CldU or IdU were given in the drinking water, rather than by injection, for 2 days at the beginning or end of the 10-day paradigm for cell labeling and behavioral testing in order to minimize handling and damage to the pump. Pilot studies confirmed similar cell labeling for both delivery methods.

### Statistical analysis, cell counts, stereology

All statistics were by Student's 2-tailed t-test or by single or multiple ANOVA with Bonferroni/Dunn post-hoc tests for multiple pair-wise comparisons using StatView software. For experiments involving many factors, F tests derived from multiple ANOVA were used to determine the main effect of single or combined variables in describing the changes and variance of the outcome measures. Quantification of histological outcomes proceeded from the null hypothesis that intervention groups would not differ bi-directionally from normal physiological controls. Cells per 40X high power field from coronal sections balanced along the a-p axis were counted for each animal and were compiled directly to obtain data points for analysis and comparison. Sections were selected objectively from each of 3 slides stereologically balanced throughout the hippocampus from each of 4 subjects per group. After testing the normality and homogeneity of the set of counts within and between subjects, the mean count per mouse was used for all group-wise comparisons. This strategy uses stereologic principles—up to the point of extrapolation—to account for the data and variability within each comparison; and assures the validity and scaling of both normal, control values and the changes from baseline observed in experimental groups. Counts were performed using a Zeiss Axiovert 200 M epifluorescent microscope and Axiovision software with appropriate blinding throughout.

### Blinding

The experimenter was kept blind to the genotype and treatment for all states of data recording, transfer and analysis. Occasionally during behavioral tests, an external fur phenotype revealed the identity of the NSE-noggin mice. For this reason, objective video tracking, automated scoring and review by a second experimenter with kappa statistic analysis was performed for all cognitive behavioral tasks.

## Supporting Information

Figure S1 Exercise exposure reduces levels of pSMAD1/5/8 in the dentate gyrus. (A–B) Two month old wild type mice were exposed to running wheels or standard housing, bounded by cell labeling with CldU and IdU according to our standard, 10-day paradigm. Coronal sections through the dentate gyrus were then immunostained for CldU (pink), pSMAD1/5/8 (red), and IdU (green). Consistent with findings in Figure 1 and with a mechanism regulating the cellular properties of the neurogenic niche, levels of pSMAD1/5/8 were reduced in and around the SGZ after 8 days of running exposure. Cells with strong nuclear (npSMAD1/5/8) staining were observed throughout the GCL in naÃ ^ve mice, but were absent in the SGZ of running mice.
(4.13 MB TIF)

Figure S2 Individual channels comprising merged images shown in Figure 2. DCX (green), CldU (pink), IdU (blue), and Sox2 (red)
(7.83 MB TIF)

Figure S3 Effects of BMP signaling on populations of progenitor cells and mature cells in the dentate gyrus. Experiments were conducted as described for Figures 2 and 3. (A–C) Unbiased stereological sampling and quantification per 40X field of cells labeled for (A) the early progenitor marker, nestin; (B) both CldU and the mature neuronal marker, NeuN; (C) the percentage of CldU-immunopositive cells also expressing NeuN 10 days after incorporation of the thymidine analog during cell division. (D–G) Coronal sections through the dentate gyrus were immunostained for DAPI (blue), NeuN (green), doublecortin (red), and CldU (pink). Panels show representative merged images from (D) Wild type, naÃ ^ve; (E) Wild type, running; (F) NSE-BMP4; (G) and NSE-noggin mice. Exposure to running and noggin increased both the number of cells expressing nestin and the number of later progenitors that divided, left the mitotic NPC pool and began to express NeuN. Noggin did not increase the proportion of CldU-labeled progenitors that later became NeuN immunoreactive. (H) Timeline for progression through the SGZ lineage, CldU labeling of late NPC division, and inception of NeuN labeling. * Differs from wild type naÃ ^ve group at p<0.01 or from other groups as indicated at p<0.03.
(10.21 MB TIF)

Figure S4 Altered BMP signaling does not regulate activity level or non-hippocampal mediated response to tones or shock. Wild type, NSE-noggin, and NSE-BMP4 mice all showed equal levels of (A) baseline activity, (B) reactivity to tone and shock and (C) extinction of conditional response during the course of a recall trial (see Methods).
(0.47 MB TIF)

Figure S5 BMP signaling influences adaptive learning on the water maze test. (A) Trial by trial scores for each day of water maze testing denoted in Figure 5C. NSE-noggin mice show notable learning between the daily primer exposure and the first trial, and also show distinct learning on a trial-by-trial basis. NSE-BMP4 mice are particularly impaired during the adaptive learning phase before the first trial, but are still able to acquire trial-by-trial spatial reference learning by the last 2 days of testing. (B) For probe tests, an additional cohort of NSE-noggin, wild type and NSE-BMP4 mice received an extra test with the target platform removed 60 minutes after the 4th target trial on training days 2–5. Upper panel: Percentage of time during the 60 second probe trial spent in the quadrant formerly containing the hidden platform. Noggin transgenic mice show a memory-based search pattern on the first probe test. Wild type mice show memory-based searching by the third probe test and NSE-BMP4 mice show probe learning on the fourth test. By the final probe trial, NSE-noggin mice spend less time in the former target quadrant during the latter half of the 60-second trial, indicative of extinction learning toward the previously rewarded response. Lower panel: The number of pass-through events during the first 15 seconds of the trial is also increased for NSE-noggin mice on the first probe test, and remains elevated for all subsequent days. NSE-BMP4 mice are also delayed in this measure of reference learning relative to wild type mice. * Differs from wild type at p<0.01 by Bonferroni/Dunn pair-wise comparison. **Significant main effect by multiple ANOVA for group x day and group x trial comparisons with all groups at p<0.05.
(0.70 MB TIF)

Figure S6 Altered Levels of BMP signaling do not affect hippocampus-independent learning or general behavior. (A) Initial performance and motor learning curves on the rotorod test show no differences in amongst NSE-noggin, wild type and NSE-BMP4 mice. (B) When provided elective access to running, all mice learn to use and continue to use the wheels equally. This confirms the effects of running in experimental groups of mice are not due differences in the distance run. (C,D) Balance beam scores for coordination and procedural learning, as measured by the time to traverse the beam (C) and the combined time to return to the home cage floor (D), are similar for each genotype.
(0.81 MB TIF)

Figure S7 Running exposure does not expand progenitor numbers or promote cell-cycle reentry across the neurogenic lineage in NSE-BMP4 mice. Quantitative analysis of immunocytochemistry for Sox2, nestin, CldU, and IdU, and double labeling of CldU+ or IdU+ cells with the early progenitor marker Sox2, for the animals described in Figure 6. Constitutively high levels of BMP4 expression prohibited the normal effects of exercise exposure to increase the numbers and rates of proliferation in early progenitor species. Student's t-test p>0.05 for all measurements.
(0.69 MB TIF)

Figure S8 Reduction of BMP signaling is not required for the effects of running on the maturation of cells at the last stages of the SGZ progenitor lineage. Groups of 2 month old NSE-BMP4 and WT mice were exposed to running or standard housing and theymidine analog labeling as described in Figures 6, S3 and S7. Coronal sections through the dentate gyrus were immunostained for DAPI (blue), NeuN (green), doublecortin (red), and CldU (pink). Panels A–D show representative merged images for all four labels; (A) Wild type, standard housing; (B) Wild type, running; (C) NSE-BMP4, standard housing; (D) NSE-BMP4, running. (E–F) Unbiased sampling and quantification of cells labeled per 40X field for all groups of mice for (E) NeuN+CldU; (F) the percentage of CldU immunopositive cells also expressing NeuN 10 days after division and thymidine analog incorporation. Running exposure increased the number of terminally dividing cells that begin to show mature neuronal labeling 10 days later. While transgenic overexpression of BMP4 was shown to reduce rates of proliferation within multiple classes of progenitor cells, it did not block running-induced increases in the proportion and the relative number of previously divided (CldU+) cells that survive and mature into NeuN+ cells. This is consistent with results in Figure S3 suggesting that down-regulation of BMP signaling reproduces the actions of exercise on mitotic progenitor populations, but not on cells exiting the NPC pool. * Differs from wild type naÃ ^ve group at p<0.01 by ANOVA and Bonferroni/Dunn pair-wise comparison.
(9.96 MB TIF)

Figure S9 Running exposure does not improve hippocampus-dependent cognition in NSE-BMP4 mice. (A–C) Two month old wild type and NSE-BMP4 mice were exposed to 8 days of running or standard housing conditions as in experiments above. At the end of the exposure, separate groups of mice were tested for hippocampus-dependent cognitive performance on the (A)Y-maze, (B) NOR or (C) NLR tests. Running improved performance in wild type mice, yet NSE-BMP4 mice showed no activity-dependent cognitive improvements.
(0.52 MB TIF)

Figure S10 Running exposure and regulation of other hippocampal protein levels in wild type and NSE-BMP4 mice. Western blot analysis for levels of BDNF shows similar duration-dependent patterns of change in wild type and NSE-BMP4 mice. Levels of PSD-95 were not affected in either group for the duration of running observed. Levels of synaptophysin were increased above baseline only in the wild type animals after 10 days of running.
(1.44 MB TIF)

Figure S11 Inhibition of cell proliferation by AraC treatment limits SGZ lineage progression in NSE-noggin mice. Additional counts and analysis of double-positive cells and histology for nestin in NSE-noggin mice infused with AraC or vehicle. (A–D) Cell cycle reentry was significantly reduced in early Sox2+ progenitors and later Dcx+ progenitors at infusion time points corresponding with cell labeling with CldU or IdU. (E) Table detailing the ratios of early or late progenitor cells undergoing cell cycle reentry at different points in time for noggin transgenic mice infused with vehicle or AraC. The number in pink on the top indicates the number of CldU+ previously divided cells per field, the number in blue on the top is the number of recently divided IdU+ cells, the number in red on the left is the number of Sox2 labeled cells, and the number in green on the left is the number of Dcx labeled cells. Black numbers indicate cells marked for division which are also labeled as a given progenitor species. Black percentages indicate the fraction of cell divisions within a given lineage species for each infusion condition. Percentages of IdU+ (dividing) cells and CldU+ (previously divided) cells expressing the later lineage marker Dcx were particularly reduced by AraC treatment. (F,G) Coronal sections through the dentate gyrus and immunohistological labeling for IdU, Dcx, nestin and CldU in NSE-noggin mice infused with vehicle or AraC. (H) Nestin+ cell counts. * Differs from vehicle control at p<0.01 by Student's t-test.
(3.38 MB TIF)

Figure S12 Additional behavioral measures and control tests for cognitive findings in Figures 7 and 8. (A) Infusion of AraC into the ventricles of NSE-noggin mice did not affect performance on the visible platform water maze control test for hippocampus-independent learning. Swim speed and general motor abilities were also not affected by infusion. (B,C) Total number of arm choices and the time elapsed during the cumulative 30 seconds of object exploration time were not different for AraC- or vehicle-infused NSE-noggin mice. (D–F) Infusion of noggin, vehicle, AraC or noggin+AraC into the ventricles of wild type mice did not affect hippocampus-independent behavior on the visually guided water maze (D), or general motor behavior on the Y-maze (E) or NOR test (F). (G–H) AraC treatment in the form of infusion of (G) AraC or noggin+AraC into wild type mice, (H) or AraC into the ventricles of NSE-noggin mice significantly impaired adaptive water maze learning on the first daily trial. However, trial-by-trial spatial reference learning, which does not depend as directly on the function of the dentate gyrus, was maintained during intraventricular AraC infusion. Similar results were obtained for NSE-BMP4 mice in Figure S5 focusing on altered properties of the DG relative to other hippocampal subregions.
(2.12 MB TIF)

Figure S13 Nestin immunohistochemistry for wild type mice infused with noggin, vehicle, AraC or noggin + AraC. Numbers of relatively quiescent, early progenitor cells expressing nestin were not significantly altered by infusion with noggin, AraC or noggin + AraC relative to vehicle controls. However wild type mice infused with noggin showed a trend toward greater numbers of nestin+cells, and mice infused with AraC or noggin + AraC showed non-significant trends toward decreased numbers. Such findings are consistent with nestin+ cells having an intermediate lineage position, spanning the populations of cells expressing Sox2 or Dcx (see Figure 8 and S3H above). These findings are also similar to those for running and transgenic animals in Figures 2, 3 and S3; yet were not as significantly different from controls, possibly owing to differences in the baseline values and variability of the two experimental systems.
(0.11 MB TIF)

Figure S14 Altered levels of the BMP target gene ID3 are spatially associated with changes in SGZ cell fate regulation in mice infused with noggin, vehicle, AraC or noggin + AraC. ID3 is a direct transcriptional target of BMP signaling that serves as a marker for levels of signaling activity in the hippocampus. Coronal sections through the dentate gyrus were immunostained for DCX (green), CldU (pink), IdU (blue), and ID3 (red). Panels A–F show representative merged images for all four labels. (A) Vehicle; (B) Noggin; (C) Vehicle at 2.5x high power; (D) Noggin at 2.5x high power; (E) AraC; (F) Noggin + AraC. Areas of reduced levels of ID3 staining correspond with locations of IdU+ cells undergoing cell cycle reentry. (G) Quantification of ID3 expression density within cells of the SGZ of noggin-infused or vehicle-infused mice. Noggin infusion increased levels of cell proliferation (Fig. 8) and reduced levels of ID3 expression in the vicinity of dividing cells (IdU+) and within later progenitors reentering cell cycle (Dcx+IdU+). Higher levels of ID3 expression in cells that divided earlier (CldU+) and then exited cell cycle were not affected by infusion. a.d.u. = normalized arbitrary density units * p<0.02 by Student's t-test.
(10.16 MB TIF)
