# Supplementary figures and images for: BMP Signaling Mediates Effects of Exercise on Hippocampal Neurogenesis and Cognition in Mice
Source: PLoS One. 2009 Oct 20;4(10):e7506. doi: 10.1371/journal.pone.0007506 (PMC2759555; doi:10.1371/journal.pone.0007506)

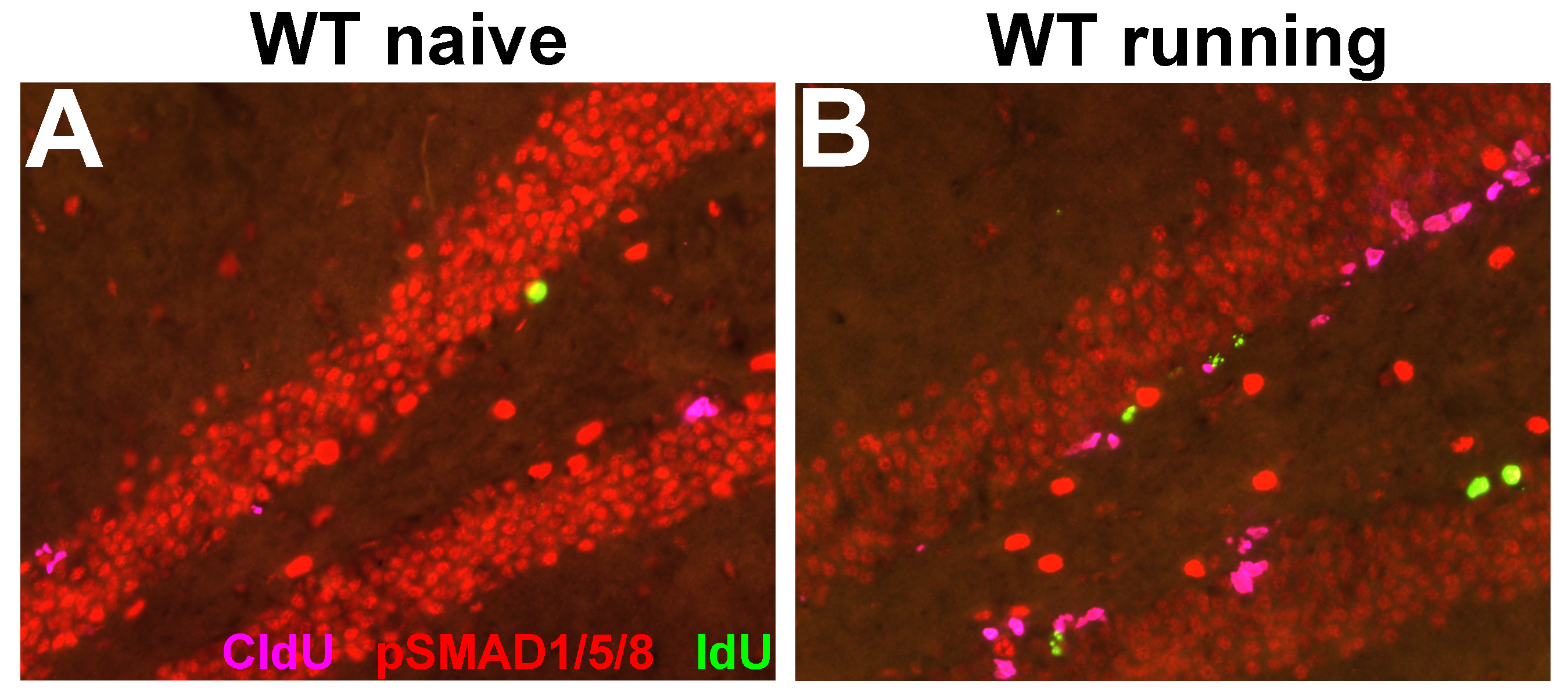

Supplement: Figure S1 — Exercise exposure reduces levels of pSMAD1/5/8 in the dentate gyrus. (A–B) Two month old wild type mice were exposed to running wheels or standard housing, bounded by cell labeling with CldU and IdU according to our standard, 10-day paradigm. Coronal sections through the dentate gyrus were then immunostained for CldU (pink), pSMAD1/5/8 (red), and IdU (green). Consistent with findings in Figure 1 and with a mechanism regulating the cellular properties of the neurogenic niche, levels of pSMAD1/5/8 were reduced in and around the SGZ after 8 days of running exposure. Cells with strong nuclear (npSMAD1/5/8) staining were observed throughout the GCL in naÃ ^ve mice, but were absent in the SGZ of running mice. (4.13 MB TIF) [file pone.0007506.s001.tif]

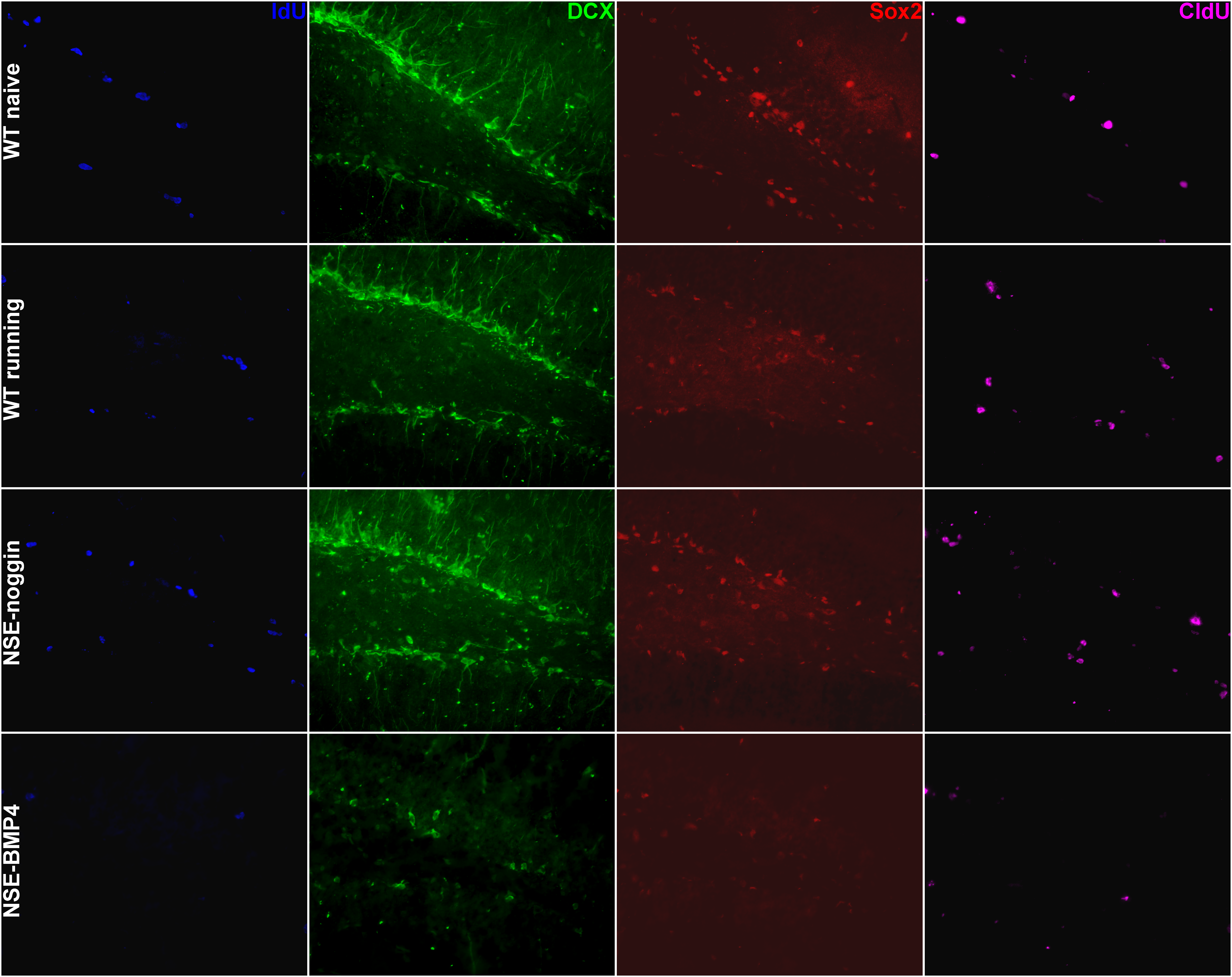

Supplement: Figure S2 — Individual channels comprising merged images shown in Figure 2. DCX (green), CldU (pink), IdU (blue), and Sox2 (red) (7.83 MB TIF) [file pone.0007506.s002.tif]

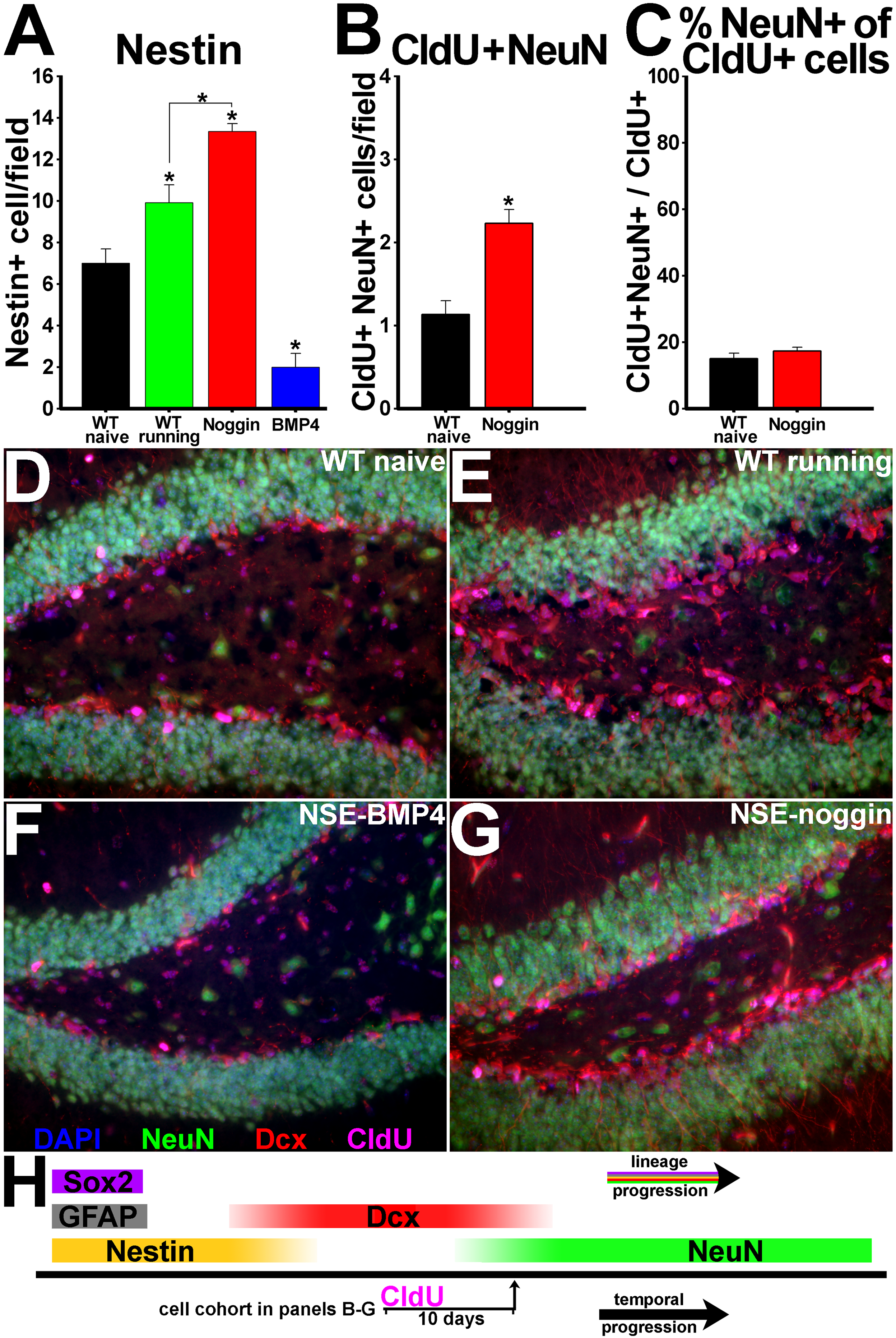

Supplement: Figure S3 — Effects of BMP signaling on populations of progenitor cells and mature cells in the dentate gyrus. Experiments were conducted as described for Figures 2 and 3. (A–C) Unbiased stereological sampling and quantification per 40X field of cells labeled for (A) the early progenitor marker, nestin; (B) both CldU and the mature neuronal marker, NeuN; (C) the percentage of CldU-immunopositive cells also expressing NeuN 10 days after incorporation of the thymidine analog during cell division. (D–G) Coronal sections through the dentate gyrus were immunostained for DAPI (blue), NeuN (green), doublecortin (red), and CldU (pink). Panels show representative merged images from (D) Wild type, naÃ ^ve; (E) Wild type, running; (F) NSE-BMP4; (G) and NSE-noggin mice. Exposure to running and noggin increased both the number of cells expressing nestin and the number of later progenitors that divided, left the mitotic NPC pool and began to express NeuN. Noggin did not increase the proportion of CldU-labeled progenitors that later became NeuN immunoreactive. (H) Timeline for progression through the SGZ lineage, CldU labeling of late NPC division, and inception of NeuN labeling. * Differs from wild type naÃ ^ve group at p<0.01 or from other groups as indicated at p<0.03. (10.21 MB TIF) [file pone.0007506.s003.tif]

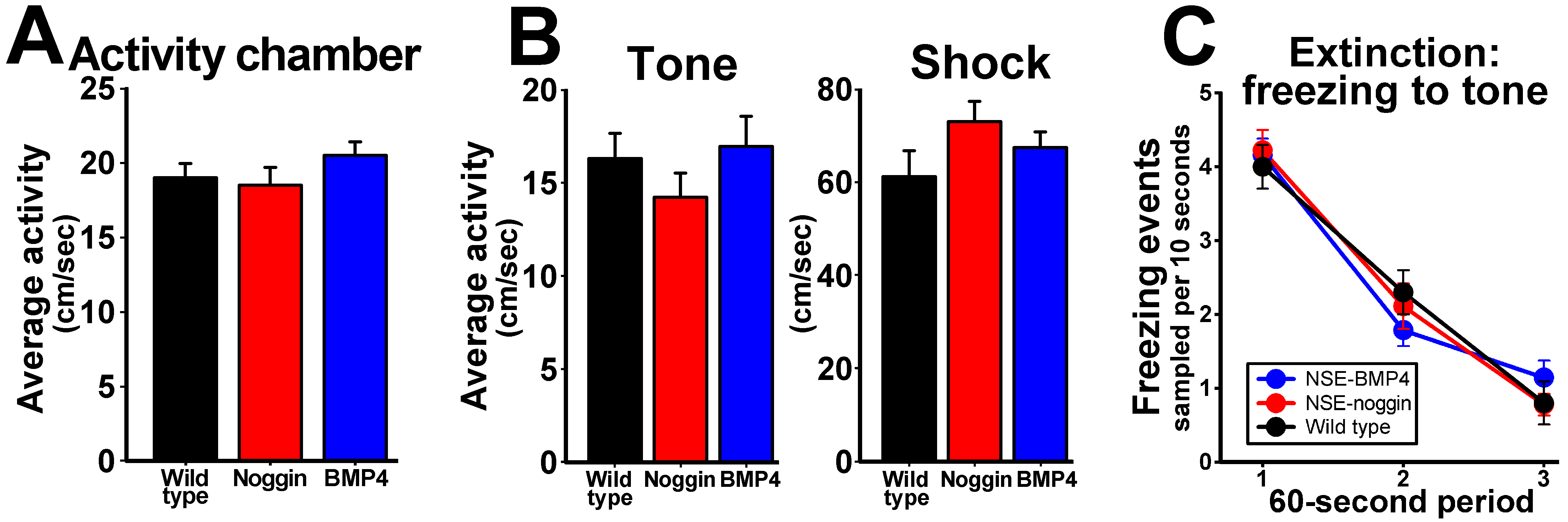

Supplement: Figure S4 — Altered BMP signaling does not regulate activity level or non-hippocampal mediated response to tones or shock. Wild type, NSE-noggin, and NSE-BMP4 mice all showed equal levels of (A) baseline activity, (B) reactivity to tone and shock and (C) extinction of conditional response during the course of a recall trial (see Methods). (0.47 MB TIF) [file pone.0007506.s004.tif]

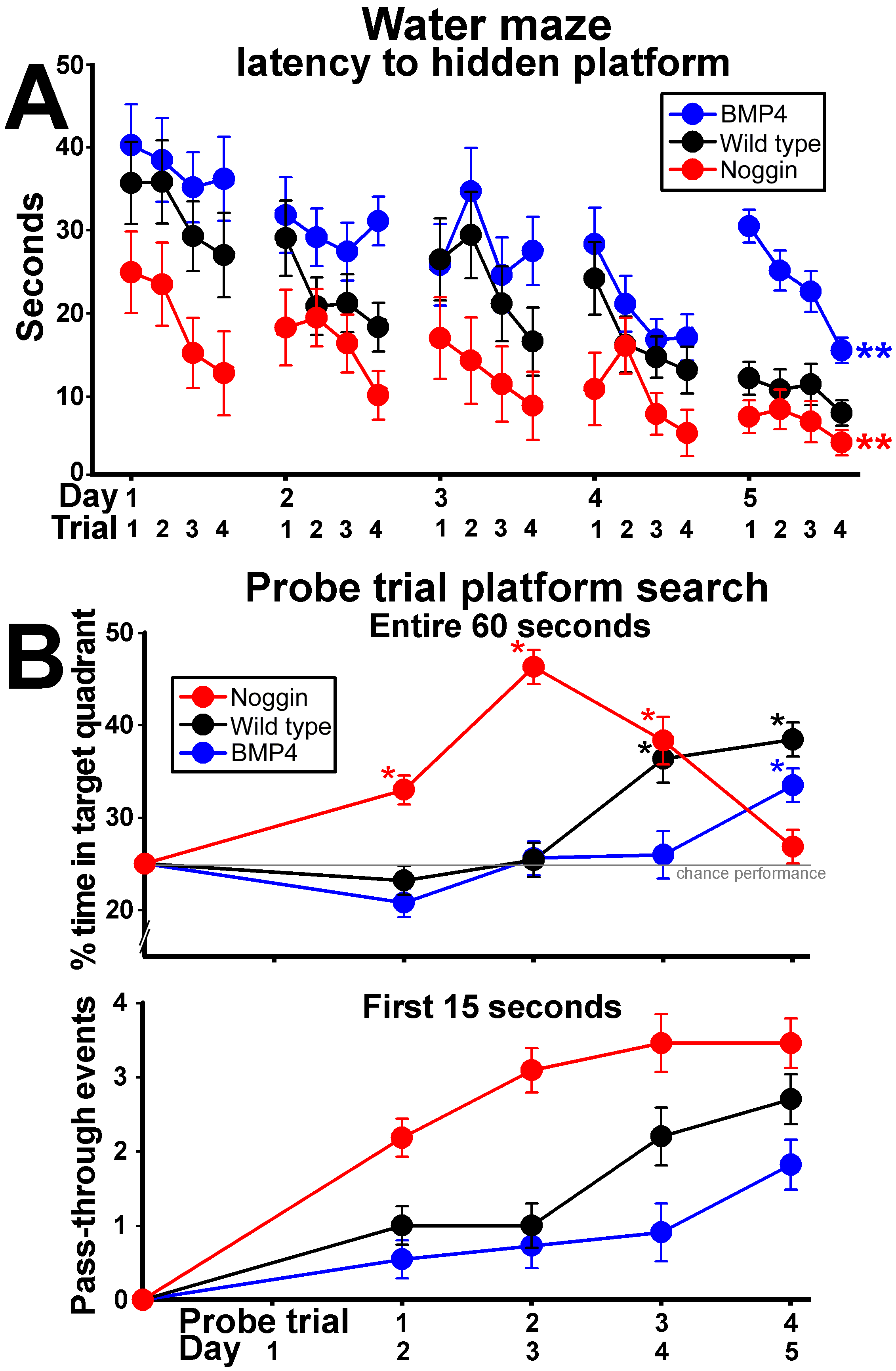

Supplement: Figure S5 — BMP signaling influences adaptive learning on the water maze test. (A) Trial by trial scores for each day of water maze testing denoted in Figure 5C. NSE-noggin mice show notable learning between the daily primer exposure and the first trial, and also show distinct learning on a trial-by-trial basis. NSE-BMP4 mice are particularly impaired during the adaptive learning phase before the first trial, but are still able to acquire trial-by-trial spatial reference learning by the last 2 days of testing. (B) For probe tests, an additional cohort of NSE-noggin, wild type and NSE-BMP4 mice received an extra test with the target platform removed 60 minutes after the 4th target trial on training days 2–5. Upper panel: Percentage of time during the 60 second probe trial spent in the quadrant formerly containing the hidden platform. Noggin transgenic mice show a memory-based search pattern on the first probe test. Wild type mice show memory-based searching by the third probe test and NSE-BMP4 mice show probe learning on the fourth test. By the final probe trial, NSE-noggin mice spend less time in the former target quadrant during the latter half of the 60-second trial, indicative of extinction learning toward the previously rewarded response. Lower panel: The number of pass-through events during the first 15 seconds of the trial is also increased for NSE-noggin mice on the first probe test, and remains elevated for all subsequent days. NSE-BMP4 mice are also delayed in this measure of reference learning relative to wild type mice. * Differs from wild type at p<0.01 by Bonferroni/Dunn pair-wise comparison. **Significant main effect by multiple ANOVA for group x day and group x trial comparisons with all groups at p<0.05. (0.70 MB TIF) [file pone.0007506.s005.tif]

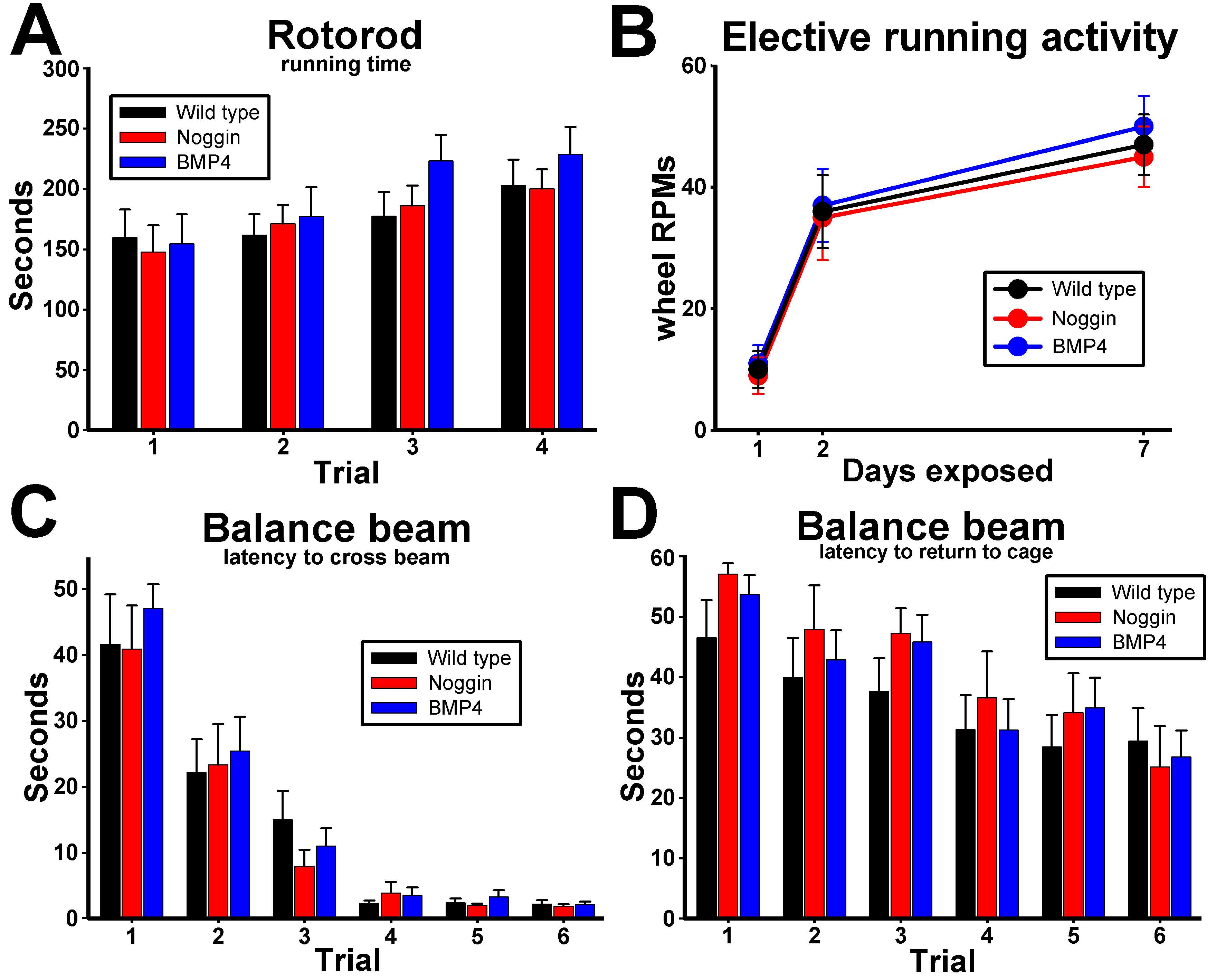

Supplement: Figure S6 — Altered Levels of BMP signaling do not affect hippocampus-independent learning or general behavior. (A) Initial performance and motor learning curves on the rotorod test show no differences in amongst NSE-noggin, wild type and NSE-BMP4 mice. (B) When provided elective access to running, all mice learn to use and continue to use the wheels equally. This confirms the effects of running in experimental groups of mice are not due differences in the distance run. (C,D) Balance beam scores for coordination and procedural learning, as measured by the time to traverse the beam (C) and the combined time to return to the home cage floor (D), are similar for each genotype. (0.81 MB TIF) [file pone.0007506.s006.tif]

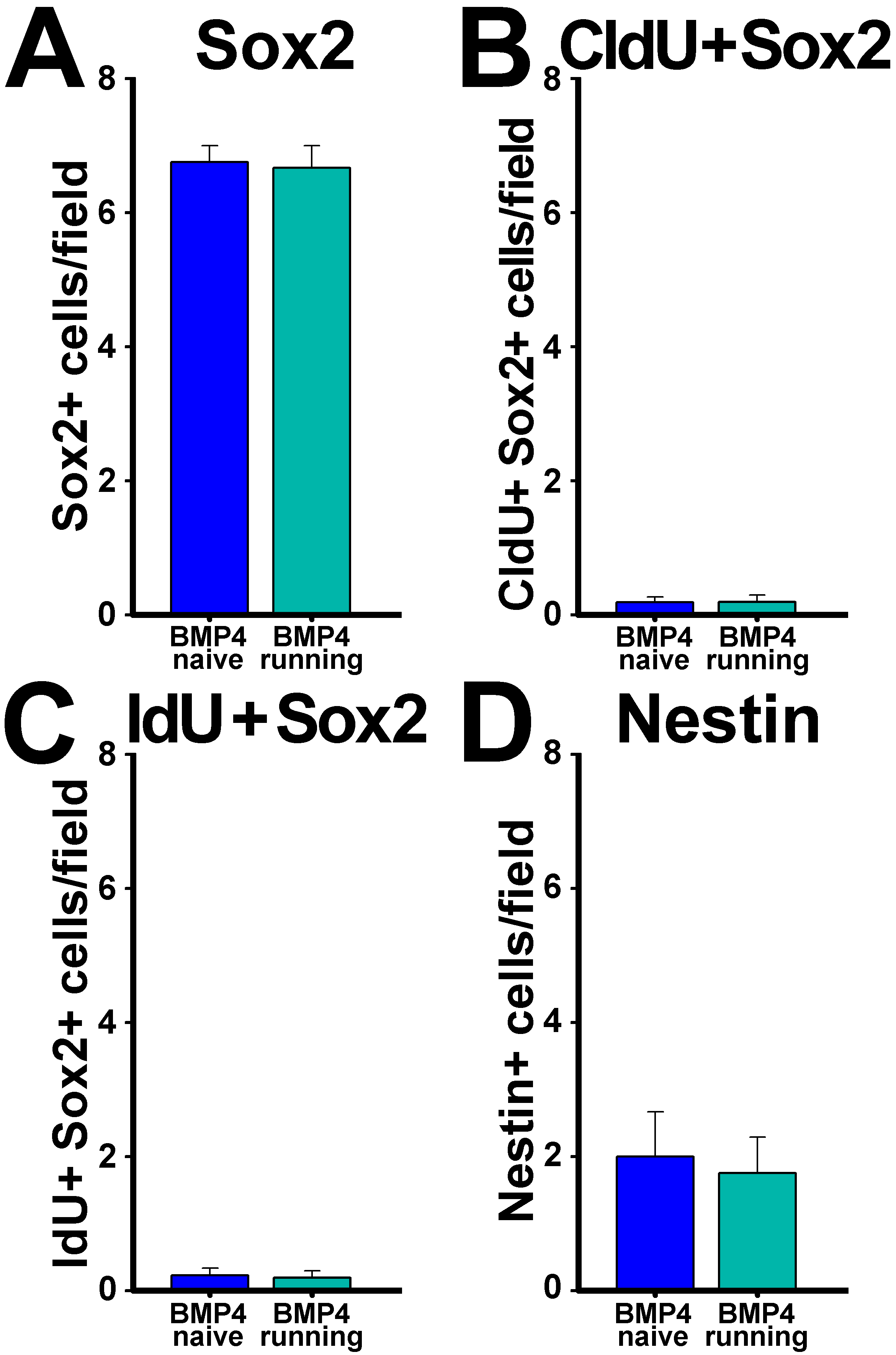

Supplement: Figure S7 — Running exposure does not expand progenitor numbers or promote cell-cycle reentry across the neurogenic lineage in NSE-BMP4 mice. Quantitative analysis of immunocytochemistry for Sox2, nestin, CldU, and IdU, and double labeling of CldU+ or IdU+ cells with the early progenitor marker Sox2, for the animals described in Figure 6. Constitutively high levels of BMP4 expression prohibited the normal effects of exercise exposure to increase the numbers and rates of proliferation in early progenitor species. Student's t-test p>0.05 for all measurements. (0.69 MB TIF) [file pone.0007506.s007.tif]

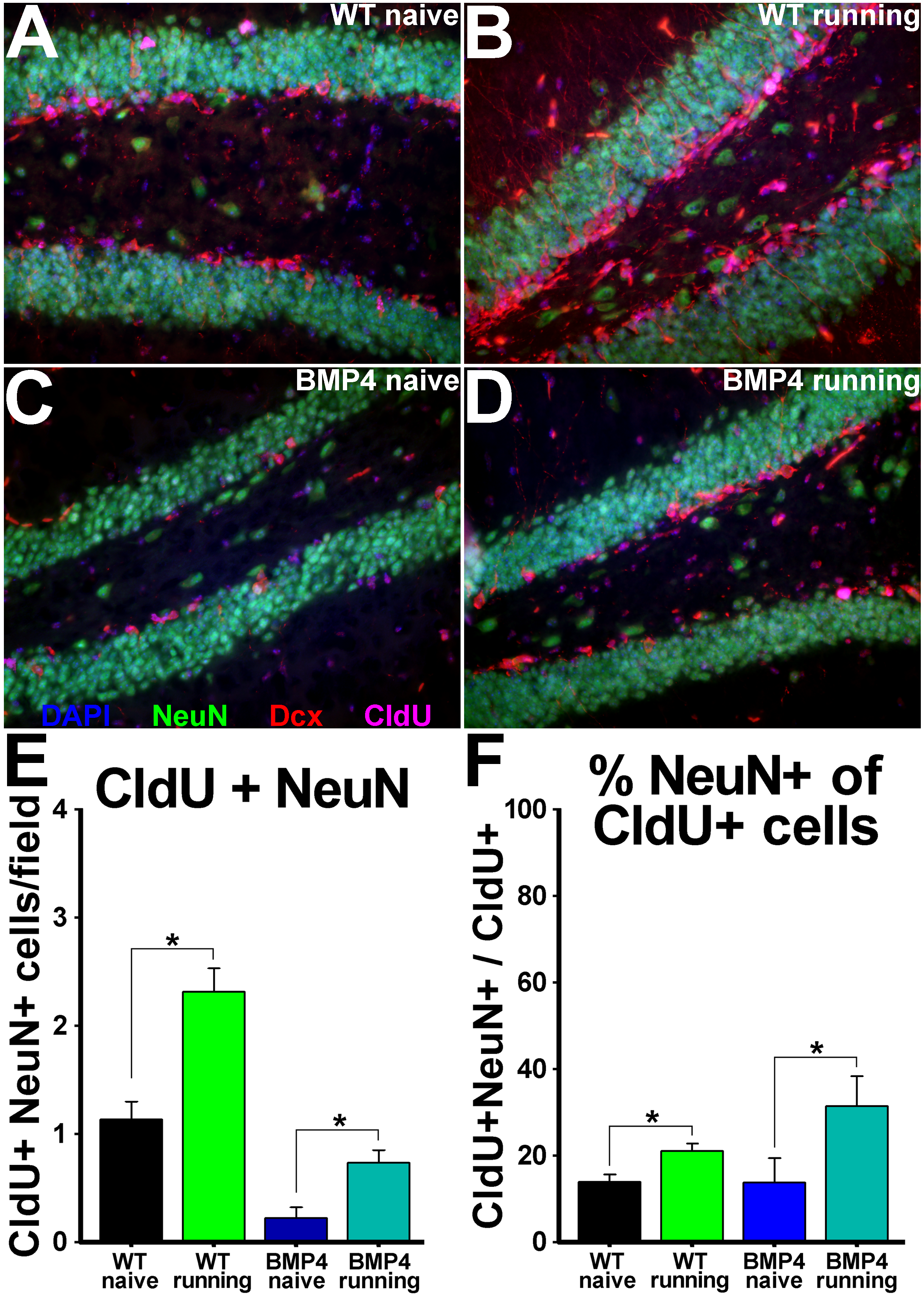

Supplement: Figure S8 — Reduction of BMP signaling is not required for the effects of running on the maturation of cells at the last stages of the SGZ progenitor lineage. Groups of 2 month old NSE-BMP4 and WT mice were exposed to running or standard housing and theymidine analog labeling as described in Figures 6, S3 and S7. Coronal sections through the dentate gyrus were immunostained for DAPI (blue), NeuN (green), doublecortin (red), and CldU (pink). Panels A–D show representative merged images for all four labels; (A) Wild type, standard housing; (B) Wild type, running; (C) NSE-BMP4, standard housing; (D) NSE-BMP4, running. (E–F) Unbiased sampling and quantification of cells labeled per 40X field for all groups of mice for (E) NeuN+CldU; (F) the percentage of CldU immunopositive cells also expressing NeuN 10 days after division and thymidine analog incorporation. Running exposure increased the number of terminally dividing cells that begin to show mature neuronal labeling 10 days later. While transgenic overexpression of BMP4 was shown to reduce rates of proliferation within multiple classes of progenitor cells, it did not block running-induced increases in the proportion and the relative number of previously divided (CldU+) cells that survive and mature into NeuN+ cells. This is consistent with results in Figure S3 suggesting that down-regulation of BMP signaling reproduces the actions of exercise on mitotic progenitor populations, but not on cells exiting the NPC pool. * Differs from wild type naÃ ^ve group at p<0.01 by ANOVA and Bonferroni/Dunn pair-wise comparison. (9.96 MB TIF) [file pone.0007506.s008.tif]

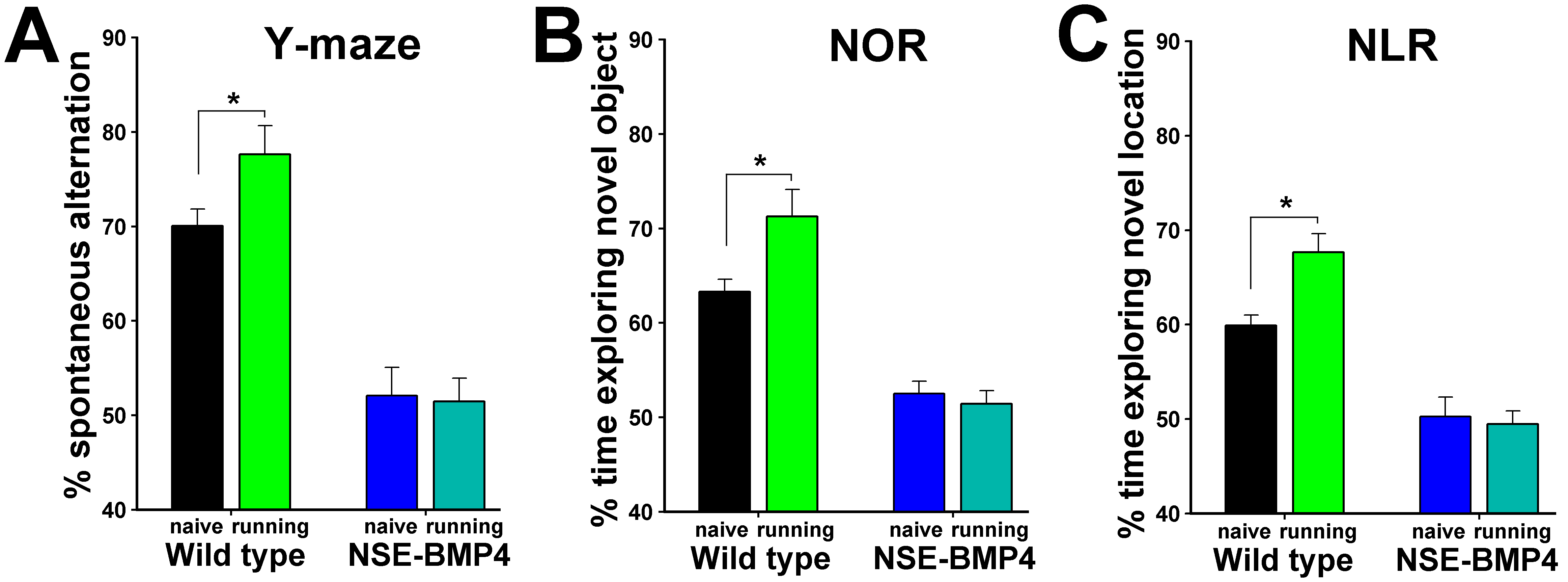

Supplement: Figure S9 — Running exposure does not improve hippocampus-dependent cognition in NSE-BMP4 mice. (A–C) Two month old wild type and NSE-BMP4 mice were exposed to 8 days of running or standard housing conditions as in experiments above. At the end of the exposure, separate groups of mice were tested for hippocampus-dependent cognitive performance on the (A)Y-maze, (B) NOR or (C) NLR tests. Running improved performance in wild type mice, yet NSE-BMP4 mice showed no activity-dependent cognitive improvements. (0.52 MB TIF) [file pone.0007506.s009.tif]

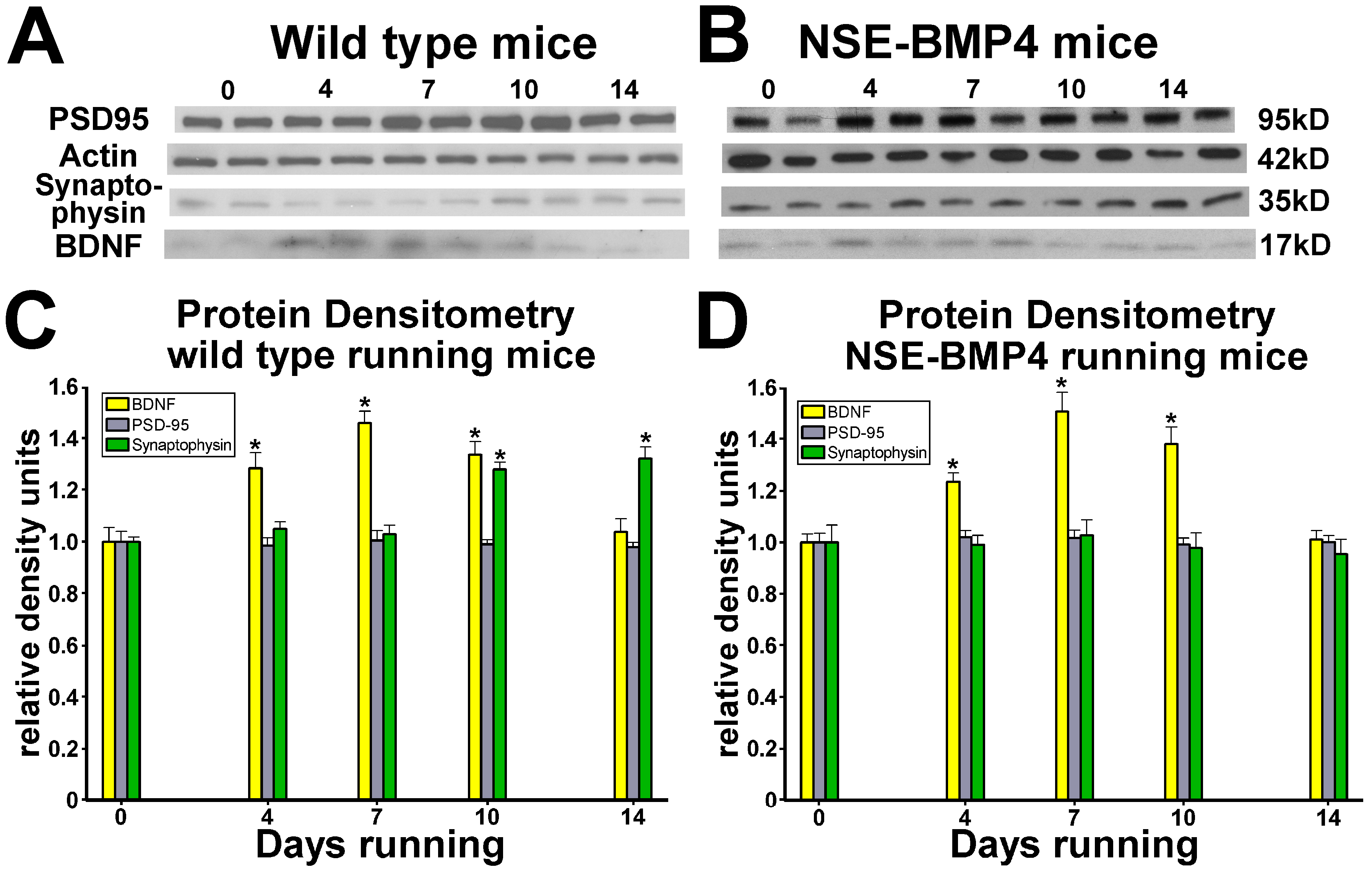

Supplement: Figure S10 — Running exposure and regulation of other hippocampal protein levels in wild type and NSE-BMP4 mice. Western blot analysis for levels of BDNF shows similar duration-dependent patterns of change in wild type and NSE-BMP4 mice. Levels of PSD-95 were not affected in either group for the duration of running observed. Levels of synaptophysin were increased above baseline only in the wild type animals after 10 days of running. (1.44 MB TIF) [file pone.0007506.s010.tif]

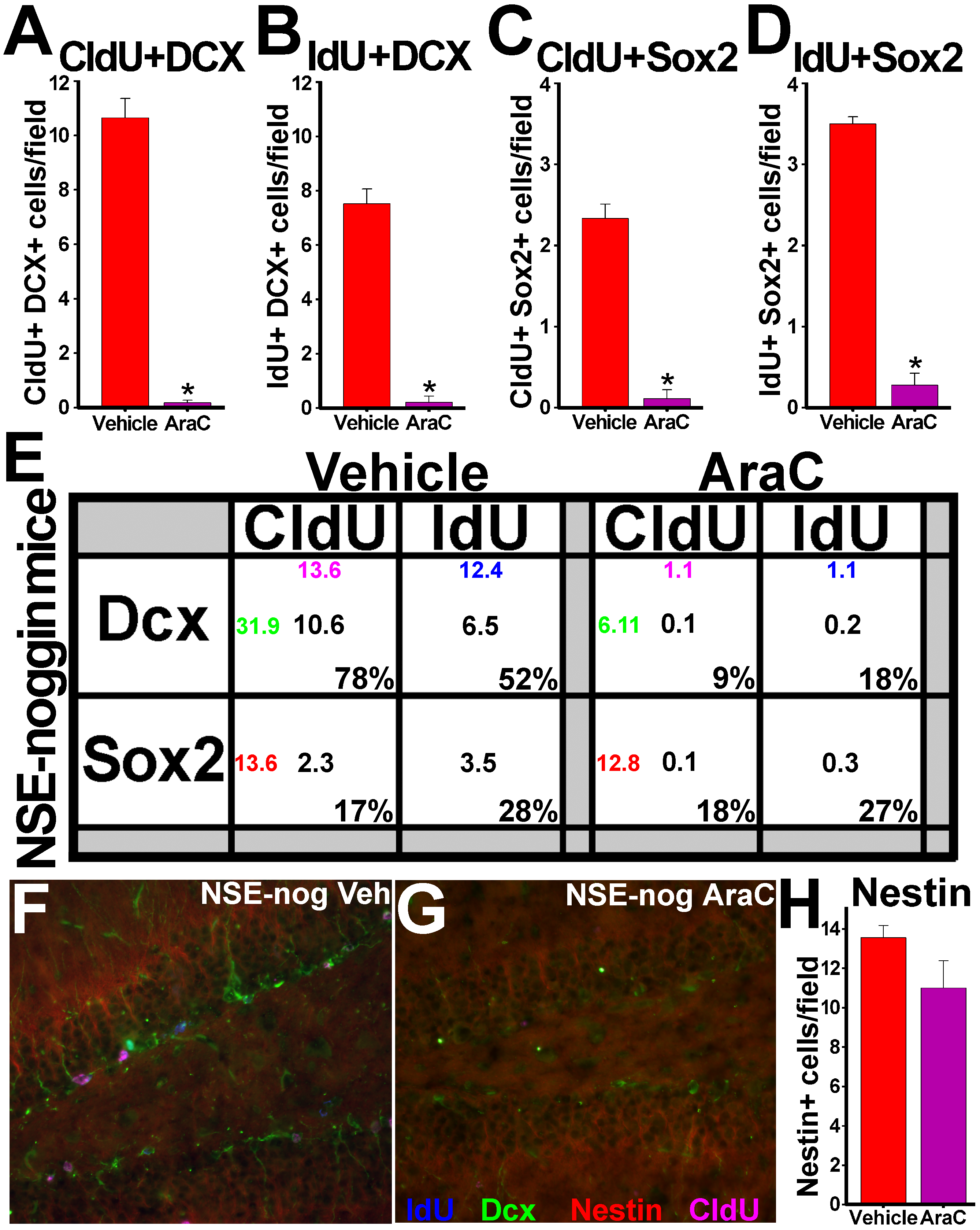

Supplement: Figure S11 — Inhibition of cell proliferation by AraC treatment limits SGZ lineage progression in NSE-noggin mice. Additional counts and analysis of double-positive cells and histology for nestin in NSE-noggin mice infused with AraC or vehicle. (A–D) Cell cycle reentry was significantly reduced in early Sox2+ progenitors and later Dcx+ progenitors at infusion time points corresponding with cell labeling with CldU or IdU. (E) Table detailing the ratios of early or late progenitor cells undergoing cell cycle reentry at different points in time for noggin transgenic mice infused with vehicle or AraC. The number in pink on the top indicates the number of CldU+ previously divided cells per field, the number in blue on the top is the number of recently divided IdU+ cells, the number in red on the left is the number of Sox2 labeled cells, and the number in green on the left is the number of Dcx labeled cells. Black numbers indicate cells marked for division which are also labeled as a given progenitor species. Black percentages indicate the fraction of cell divisions within a given lineage species for each infusion condition. Percentages of IdU+ (dividing) cells and CldU+ (previously divided) cells expressing the later lineage marker Dcx were particularly reduced by AraC treatment. (F,G) Coronal sections through the dentate gyrus and immunohistological labeling for IdU, Dcx, nestin and CldU in NSE-noggin mice infused with vehicle or AraC. (H) Nestin+ cell counts. * Differs from vehicle control at p<0.01 by Student's t-test. (3.38 MB TIF) [file pone.0007506.s011.tif]

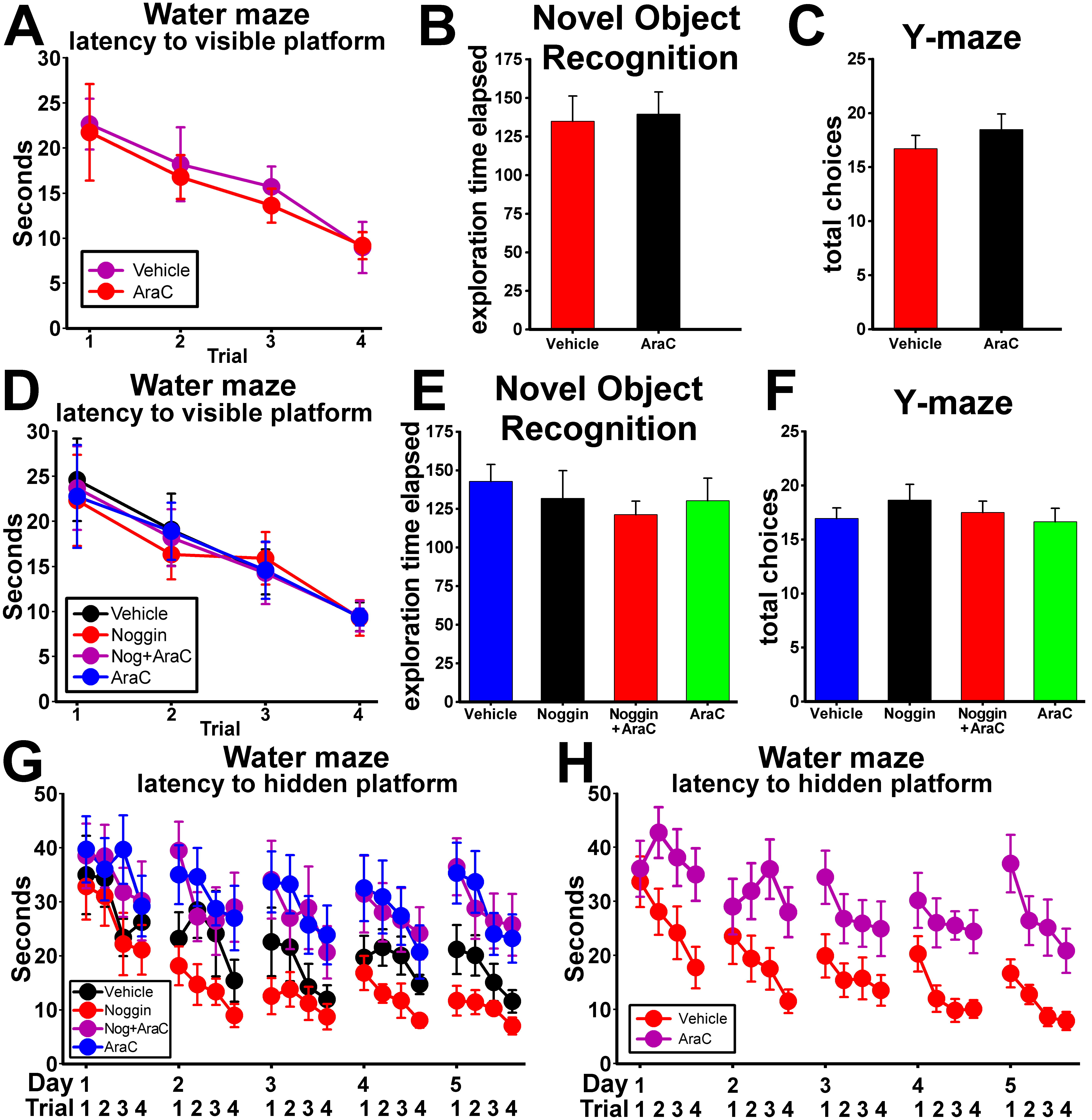

Supplement: Figure S12 — Additional behavioral measures and control tests for cognitive findings in Figures 7 and 8. (A) Infusion of AraC into the ventricles of NSE-noggin mice did not affect performance on the visible platform water maze control test for hippocampus-independent learning. Swim speed and general motor abilities were also not affected by infusion. (B,C) Total number of arm choices and the time elapsed during the cumulative 30 seconds of object exploration time were not different for AraC- or vehicle-infused NSE-noggin mice. (D–F) Infusion of noggin, vehicle, AraC or noggin+AraC into the ventricles of wild type mice did not affect hippocampus-independent behavior on the visually guided water maze (D), or general motor behavior on the Y-maze (E) or NOR test (F). (G–H) AraC treatment in the form of infusion of (G) AraC or noggin+AraC into wild type mice, (H) or AraC into the ventricles of NSE-noggin mice significantly impaired adaptive water maze learning on the first daily trial. However, trial-by-trial spatial reference learning, which does not depend as directly on the function of the dentate gyrus, was maintained during intraventricular AraC infusion. Similar results were obtained for NSE-BMP4 mice in Figure S5 focusing on altered properties of the DG relative to other hippocampal subregions. (2.12 MB TIF) [file pone.0007506.s012.tif]

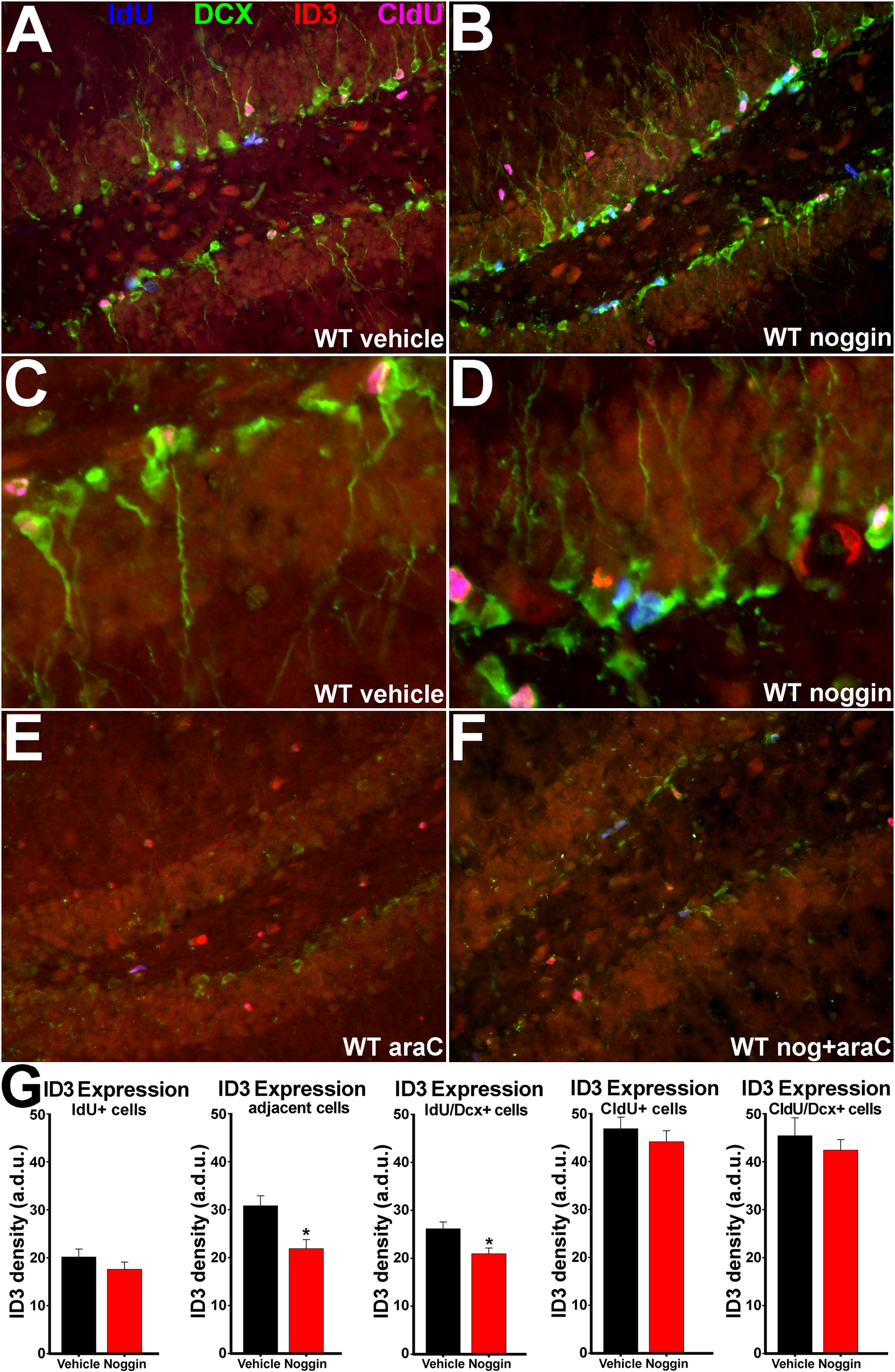

Supplement: Figure S14 — Altered levels of the BMP target gene ID3 are spatially associated with changes in SGZ cell fate regulation in mice infused with noggin, vehicle, AraC or noggin + AraC. ID3 is a direct transcriptional target of BMP signaling that serves as a marker for levels of signaling activity in the hippocampus. Coronal sections through the dentate gyrus were immunostained for DCX (green), CldU (pink), IdU (blue), and ID3 (red). Panels A–F show representative merged images for all four labels. (A) Vehicle; (B) Noggin; (C) Vehicle at 2.5x high power; (D) Noggin at 2.5x high power; (E) AraC; (F) Noggin + AraC. Areas of reduced levels of ID3 staining correspond with locations of IdU+ cells undergoing cell cycle reentry. (G) Quantification of ID3 expression density within cells of the SGZ of noggin-infused or vehicle-infused mice. Noggin infusion increased levels of cell proliferation (Fig. 8) and reduced levels of ID3 expression in the vicinity of dividing cells (IdU+) and within later progenitors reentering cell cycle (Dcx+IdU+). Higher levels of ID3 expression in cells that divided earlier (CldU+) and then exited cell cycle were not affected by infusion. a.d.u. = normalized arbitrary density units * p<0.02 by Student's t-test. (10.16 MB TIF) [file pone.0007506.s014.tif]
